# HVIface: sequence-based deep learning for decoding human-virus protein-protein interfaces

**DOI:** 10.3389/fbinf.2026.1813796

**Published:** 2026-05-08

**Authors:** Krishna Kant Gupta, Monty Vijayvargiya, Geetha Paul, Ninan Sajeeth Philip, Radha Chauhan

**Affiliations:** 1 Laboratory of Macromolecular Assemblies and Dynamics, Lab 02 New Building, National Center for Cell Science, Pune, India; 2 School of Chemical and Biotechnology, SASTRA University, Thanjavur, Tamil Nadu, India; 3 Regional Centre for Biotechnology, Faridabad, India; 4 Artificial Intelligence Research and Intelligent Systems, Thelliyoor, Kerala, India

**Keywords:** ANN, deep-learning, integrase, network analysis, Nup62, protein-protein interactions

## Abstract

**Introduction:**

Human–virus protein–protein interactions (PPIs) govern critical steps in viral entry, replication, and immune modulation. Despite their importance, the physicochemical determinants underlying human–virus interface recognition remain incompletely understood. A detailed characterization of these determinants is essential for uncovering viral hijacking mechanisms and enabling therapeutic targeting. To address this, we developed a sequence-based deep learning framework, HVIface, for residue-level prediction of human–virus PPI interfaces.

**Methods:**

HVIface was trained on a curated dataset of 73 structurally resolved human–virus complexes obtained from the Protein Data Bank. The model integrates 18 sequence-derived features, including residue contact potentials, physicochemical compatibility measures, and co-evolutionary signals. Performance was evaluated on an independent test set and compared against existing methods such as PIPENN and PeSTo. Additionally, feature importance analysis was conducted to identify key determinants contributing to interface prediction.

**Results:**

HVIface achieved an accuracy of 0.85 on the independent test set, outperforming existing approaches in identifying human–virus interface residues. Feature importance analysis highlighted environmentally weighted charge compatibility and co-evolution–derived contact potentials as dominant contributors. Case studies involving HIV-1 Integrase–Nup62, Influenza PB1–Nup85, and HCV NS5A–LASP1 complexes demonstrated that viral proteins preferentially target solvent-accessible, electrostatically favorable host regions through compact hotspot-driven architectures.

**Discussion:**

The findings reveal that electrostatic complementarity and localized residue clustering are central to viral–host interface formation, suggesting conserved physicochemical principles underlying viral targeting strategies. HVIface not only provides a robust predictive framework but also offers mechanistic insights into human–virus interface chemistry. These insights can facilitate systematic exploration of viral interactions and support the development of targeted therapeutic interventions. The source code is available at: https://github.com/krishnakantgupta-ai/HVIface_MMAD.

## Highlights

- HVIface is a sequence-based deep learning framework for residue-level prediction of human–virus protein–protein interfaces.
- The model achieves 85% accuracy on independent test complexes and demonstrates robust generalization across 16 validation datasets.
- Case studies of Nup62–HIV-1 Integrase, LASP1–NS5A, and Nup85–PB1 reveal compact, electrostatically driven hotspot architectures, with the Nup62–Integrase interaction further supported by experimental validation.
- HVIface outperforms existing predictors, including PIPENN and PeSTo, in identifying human–virus interface residues.

## 1 Introduction

Viruses pose a persistent global health threat by exploiting host cellular machinery to replicate, disseminate, and evade immune surveillance. Although the sequence of viral attachment, entry, genome replication, and host modulation varies across viral families, these processes are governed by conserved molecular principles rooted in viral–host protein interactions (Howley et al., 2022). Deciphering these interactions is essential for understanding disease mechanisms and for developing targeted antiviral therapeutics and vaccines (V’kovski et al., 2021). The COVID-19 pandemic further underscored the urgency of systematically characterizing viral infection pathways and their molecular determinants (Mallah et al., 2021). Central to viral pathogenesis are protein–protein interactions (PPIs) between viral and host proteins, through which viruses reprogram cellular pathways to promote replication and persistence (Koonin et al., 2022; Li et al., 2022). However, identifying the precise residue-level interfaces that mediate these interactions remains a major unresolved challenge. Human–virus protein–protein interaction interfaces (PPIIs) are often transient, structurally heterogeneous, and dynamically regulated, complicating their experimental and computational characterization.

Large-scale host–virus interaction datasets generated using yeast two-hybrid screening (Lin et al., 2017), mass spectrometry (Richa et al., 2021), and TAP-tagging (Goodfellow et al., 2014) have enabled the development of machine learning (ML) and deep learning (DL) (Soleymani et al., 2022) approaches for predicting PPIs. Nevertheless, many experimental techniques, such as co-immunoprecipitation, provide only domain-level or interaction-level information, whereas atomic-resolution interface mapping requires cryo-electron microscopy or X-ray crystallography, both of which are technically demanding and resource-intensive (Lu et al., 2025). Early computational strategies relied on Support Vector Machines (SVMs) and Random Forests (RFs), including models such as those developed by Guo et al. (2008), PPI-Detect (Romero-Molina et al., 2019), ACTSVM (Ma et al., 2020), and related approaches (Ahmed et al., 2018), as well as RF-based methods incorporating Minimum Description Length features (You et al., 2015). Our previously developed CoRNeA pipeline (Chopra et al., 2020) integrates co-evolutionary signals, random forest learning, and network analysis to improve eukaryotic interface prediction. More recently, deep learning architectures—including RNNs, CNNs, and transformers—have advanced virus–host PPI prediction (Lian et al., 2021), exemplified by DeepViral (Liu-Wei et al., 2021). In parallel, curated databases such as HCVpro (Kwofie et al., 2011), DenHunt (Karyala et al., 2016), DenvInt (Dey and Mukhopadhyay, 2017), ZikaBase (Gurumayum et al., 2018), VirHostNet (Navratil et al., 2009), VirusMentha (Calderone et al., 2015), VirusMINT (Chatr-aryamontri et al., 2009), and HVIDB (Yang et al., 2021) provide valuable interaction-level resources. Despite these developments, comprehensive residue-level annotations of human–virus PPIs remain limited, restricting mechanistic insight into interface chemistry.

**FIGURE 1:**
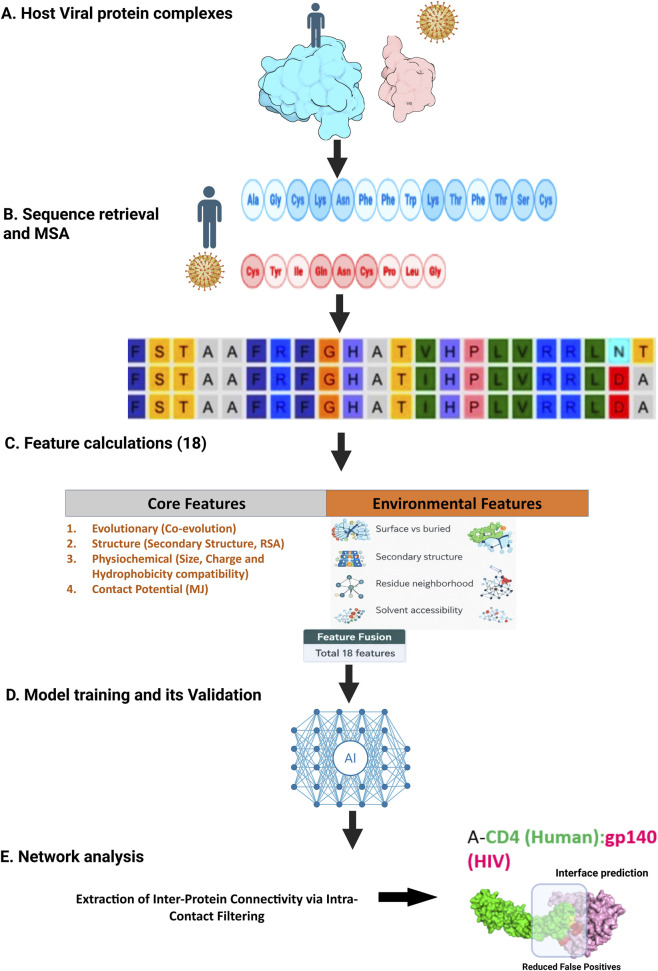
Workflow diagram illustrating five steps: **(A)** host viral protein complex formation; **(B)** sequence retrieval and multiple sequence alignment; **(C)** calculation of 18 core and environmental features; **(D)** AI model training and validation; **(E)** network analysis with inter-protein connectivity extraction, resulting in interface prediction between CD4 (human, green) and gp140 (HIV, pink).

To address this gap, we developed HVIface (Figure 1), a sequence-based deep learning framework for residue-level prediction of human–virus protein interfaces. HVIface is trained on 73 experimentally resolved human–virus complex structures and leverages 18 CoRNeA-derived features that capture contact potentials, physicochemical compatibility, and co-evolutionary relationships. Due to the limited availability of high-resolution, experimentally resolved human–virus protein complexes with residue-level interface annotation, the dataset size remains inherently constrained. Therefore, we curated a non-redundant, high-quality dataset to ensure biological relevance and reliable interface labeling. Following a complex-level splitting strategy, entire protein complexes (rather than individual residue pairs) were assigned exclusively to either the training or test sets, ensuring that no information from a given complex is shared between the two. This approach prevents data leakage and enables a more rigorous evaluation of model generalization. Using this strategy, 80% of the complexes were used for training. To evaluate real-world applicability, we further examined three biologically relevant complexes—HIV-1 Integrase–Nup62, Influenza PB1–Nup85, and HCV NS5A–LASP1—and benchmarked HVIface against two contemporary deep learning predictors, PeSTo (Krapp et al., 2023) and PIPENN (Stringer et al., 2022). Across 16 independent datasets, HVIface demonstrated consistently improved interface prediction performance.

Collectively, HVIface is a deep learning–based extension of the CoRNeA framework, specifically redesigned for predicting human–virus protein–protein interaction interfaces. While inspired by CoRNeA, HVIface incorporates substantial modifications in dataset construction, model architecture, and feature design to address the unique challenges of inter-species interactions.

## 2 Data and methods

### 2.1 HVIface workflow

The CoRNeA pipeline was employed with the exception of the artificial neural network (ANN) module. Paired contact-forming residues were predicted in three stages: (i) pairwise features-including structural descriptors, contact potentials and co-evolution scores, are derived from amino acid sequences; (ii) features are processed through a deep learning ANN with optimized hyperparameters; and (iii) intra-protein contacts inferred from co-evolution are integrated with ANN-predicted inter-protein contacts to identify interface residues, followed by network analysis (Figure 1).

### 2.2 Datasets

Human-virus protein complexes were compiled from the PDB, covering 14 viruses, including Vaccinia, Cytomegalovirus, Epstein-Barr, Nipah, HCV, HIV, Influenza, and SARS-CoV-2. Structures with resolution ≤3.0 Å were selected. Protein complexes containing antibodies were not included in this analysis due to the unavailability of homologous sequences. Sequences were retrieved from UniProt (The UniProt Consortium, 2023) and used to query PHMMER (Finn et al., 2011) for homologs, followed by redundancy removal and filtering at ≥25% identity. Only protein sequences longer than 40 amino acids were retained. Structure-guided MSAs were generated using PROMALS3D (Pei et al., 2008), refined in JalView (v 2.11.0) (Waterhouse et al., 2009), and concatenated in R (v 4.2.3) to compute co-evolutionary matrices for interface analysis.

A total of host–virus protein complex structures were initially retrieved from publicly available databases. From this dataset, complexes corresponding to 14 viral species were selected based on availability and structural quality. To ensure data consistency, filtering criteria were applied, including removal of redundant entries, incomplete structures, and complexes lacking sufficient interface annotation. After filtering, a total of 73 non-redundant protein complexes were retained for further analysis.

### 2.3 Feature generation

**FIGURE 2:**
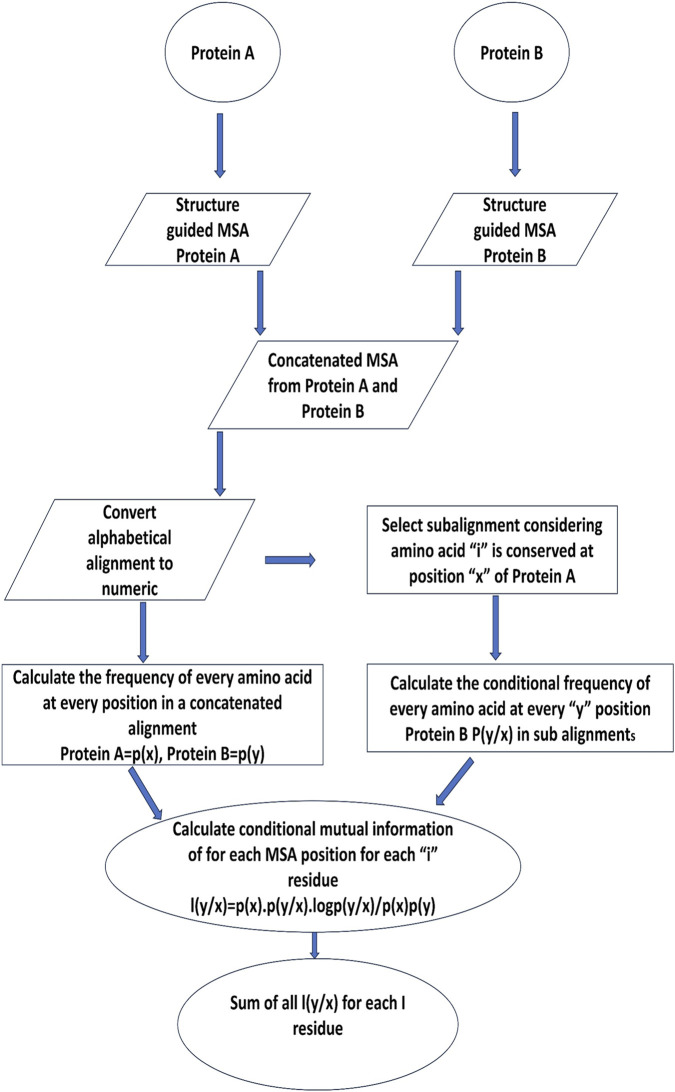
An approach for determining inter-protein co-evolving sites from multiple sequence alignments is shown in a flow chart.

The features included co-evolution scores from concatenated MSAs (Statistical Coupling Analysis and conditional mutual information) (Figure 2) (Teşileanu et al., 2015; Zan et al., 2022), physicochemical compatibility matrices (size, charge, hydropathy), relative solvent accessibility (RSA) from SABLEII, secondary structure from PSIPRED (McGuffin et al., 2000), and three Miyazawa-Jernigan contact potential matrices (Krissinel and Henrick, 2007). Environmental context was incorporated by convolving each feature matrix with a 5 × 5 kernel, producing 18 matrices per protein pair. All features were normalized between 0 and 1 (Supplementary Figure S1).

### 2.4 Interface labelling

The interface residues within a 10 Å distance cutoff were annotated using Interfaceresidues. py, InterCAAT (Grudman et al., 2022), and PDBePISA. Labels were generated with a custom Python script (labellingcodefurther.py; Supplementary Material.doc). A relatively permissive distance cutoff of 10 Å was used to capture not only direct residue–residue contacts but also proximal residues contributing to the interaction environment, including electrostatic and structural context, which are important for sequence-based learning.

The numbers in the script arise from the internal indexing used during model training and prediction, where residue–residue interactions are represented in a flattened M × N matrix. The offsets (49 for human protein and 83 for viral protein) correspond to the starting residue indices used to align predicted interaction positions back to the original protein sequences.

### 2.5 Data split and ANN architecture

The ANN was implemented in Keras v (3.12.0) with hyperparameters detailed in the Supplementary Material.doc. Our dataset consists of 73 independent protein complexes, each stored as a separate CSV file. The 80/20 split was performed at the complex level (i.e., file-wise splitting), not at the residue-pair level within the same complex. Thus, all residue pairs belonging to a given complex were kept exclusively within either the training or the test set (https://github.com/krishnakantgupta-ai/HVIface_MMAD/blob/main/Models_Scripts/Final_model_development_15_04_2024.py). Class imbalance in the training set was addressed using the *imblearn* library by evaluating multiple strategies, including undersampling, oversampling, SMOTE, and hybrid approaches such as K-means–Tomek links. While combined sampling methods improved class balance, SMOTE yielded the most favorable performance and was therefore selected for the final model.

The model was trained for 50 epochs with early stopping, using the Adam optimizer (learning rate 0.001) and binary cross-entropy loss. The important features were evaluated using the SelectKBest method. Although the top seven features were sufficient, the final model was trained using all 18 features. The trained HVIface model was evaluated on 16 test protein complexes, and its performance metrics, including the confusion matrix, were computed on this independent test set.

To assess the robustness and generalizability of the model, 10-fold cross-validation was performed. The dataset was divided into 10 subsets based on unique protein complexes. In each fold, 9 subsets were used for training and the remaining subset was used for testing. Performance metrics including accuracy and receiver operating characteristic area under the curve (ROC-AUC) were computed for each fold, and the mean ± standard deviation across all folds was reported.

To evaluate cross-family generalization, a Leave-One-Family-Out (LOFO) validation strategy was implemented. Viral families were assigned to each complex based on filename prefixes (e.g., HIV, Influenza, SARS-CoV-2). In each iteration, all complexes belonging to one viral family were excluded from the training set and used exclusively for testing, while the model was trained on complexes from the remaining families. This approach assesses the ability of the model to generalize to unseen viral families.

### 2.6 Network-based refinement and benchmarking of HVIface interface predictions

**FIGURE 3:**
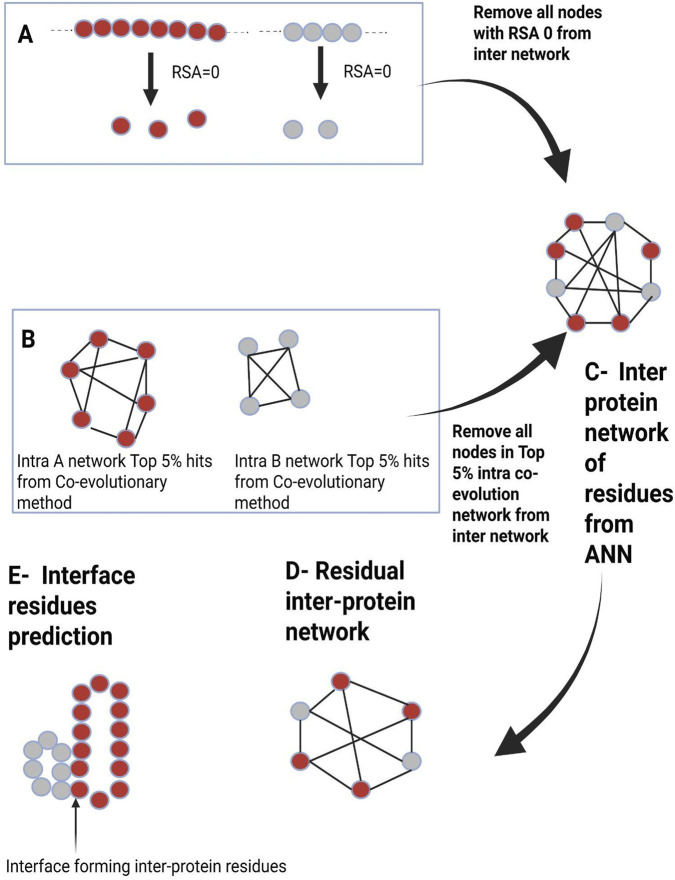
Network analysis of interactions between and within proteins. **(A)** Protein A and B residue extraction with relative solvent accessibility (RSA) = 0. **(B)** Protein A and B intra-contact prediction (top 5% co-evolving residue pairs). **(C)** Artificial Neural Network-predicted interprotein network. **(D)** By excluding nodes with RSA = 0 for Protein A and B and the top 5% co-evolving intra-protein residues of Protein A and B, the false-positive inter-protein residue pairs derived from the Random Forest classifier are decreased. **(E)** Examination of the residual network’s intercontact with Protein A and B’s structures. Using RStudio’s igraph package, the inter-protein residue network was plotted as a bipartite graph based on the ANN predictions. These residues were retrieved using SABLEII for both proteins separately because the RSA for residues found in the protein’s core should be 0. Thus, after removing nodes corresponding to RSA = 0 and intra-protein contacts from Proteins A and B, a residual network was constructed for inter-protein contact prediction.

The intra-protein contacts were computed by concatenating each protein with itself and selecting the top 5% co-evolution scores after removing residue pairs separated by < 5 positions. Residue networks were visualized with igraph. Inter-protein predictions were arranged in a binary M × N matrix, with 1 and 0 representing predicted interface and non-interface pairs. A 3 × 3 or 5 × 5 convolution kernel was applied to score local clusters, and high-confidence interface residues were extracted based on thresholds of 2 (3 × 3) or 6 (5 × 5) for mapping onto test-set structures (Figure 3). The cutoff values 2 and 6 were chosen based on the density of predicted positives within local windows (3 × 3 and 5 × 5)-specifically, representing at least ∼25% positive occupancy. Also, the evaluation metrics of all 16 test cases after network analysis were assessed and reported. The predicted interfaces of the PDB IDs 4U0A, 4U0C, and 1Q94 in the test cases were assessed and reported.

### 2.7 Case study evaluation

#### 2.7.1 Modelling and docking of human-virus complexes

Previous studies have reported interactions between LASP1–NS5A (Choi et al., 2020) and Nup85–PB1 (Ling et al., 2022). The experimental structure of LASP1 (PDB ID 3I35) was considered for docking with the modelled structure of HCV NS5A. Similarly, the Human Nup85 (PDB ID 5A9Q) was considered for docking with the modelled structure of the influenza virus PB1. These proteins were modelled using AlphaFold3. The blind flexible docking was then performed using HDOCK, and the resulting interface residues were compared with those predicted by the HVIface model. These analyses were conducted solely for validation purposes and were not included in model training or testing. The predicted structures were assessed using pLDDT scores, with interface regions predominantly corresponding to high-confidence residues.

#### 2.7.2 Nup62-HIV-1 integrase interaction

The experimentally resolved human Nup62 structure (PDB ID: 5IJN), in both monomeric and dimeric forms, was used in combination with the HIV-1 Integrase (UniProt: A0A0U2V1G2), which was modelled using AlphaFold3 (Ao et al., 2012; Abramson et al., 2024). The predicted complexes were subjected to blind flexible docking using HDOCK (Yan et al., 2017), and interface residues were analyzed. These case studies were used to qualitatively assess the predictive performance of HVIface. Structural confidence was evaluated using pLDDT scores, confirming that the interacting regions are largely associated with high-confidence predictions.

The C-terminal coiled-coil domain of human Nup62 (residues 322–522) was cloned into pRSFDuet-1 with an N-terminal 6XHis tag, and full-length HIV Integrase was cloned into pGEX6P1. Both plasmids were co-transformed into *E. coli* Rosetta (DE3) pLysS RIL cells via heat shock, induced with 0.4 mM IPTG at 20 °C overnight, and harvested after 14-16 h. Cells were lysed in buffer (25 mM HEPES, pH 7.4; 200 mM NaCl; 2 mM BME; 1 mM PMSF) by ice incubation, sonicationand centrifugationand the supernatant was incubated with glutathione beads for 4 h. After three washes with lysis buffer, bound proteins were eluted in a buffer containing 25 mM HEPES (pH 7.4), 200 mM NaCl, 2 mM BME, 10% glycerol and 10 mM reduced glutathione. Eluted fractions were resolved on 12% SDS-PAGE, transferred to PVDF membranes, and probed with Anti-Nup62 (1:3000) and Anti-GST (1:3000), followed by HRP-conjugated secondary antibody (1:5000). Blots were developed using the Quant HRP substrate and imaged on an Amersham Imager 600.

### 2.8 Comparison with PeSTo and PIPENN

The PeSTo tool represents proteins as translation-invariant point clouds with atomic coordinates and element types, updated via geometric transformers. PDB assemblies clustered at 30% identity were split into training (70%), validation (15%), and testing (15%), with interfaces defined by contacts within 5 Å. The PIPENN tool’s features included amino acid type, sequence length, domain, conservation (PSSM), predicted secondary structure, and RSA/ASA, with local-context features averaged over 3-9 residue windows (128 features total). The sixteen test complexes were used to compare PeSTo and PIPENN predictions with HVIface using a 5 Å cutoff to define direct residue–residue contacts, consistent with standard structural definitions and with the criteria used by both PeSTo and PIPENN.

## 3 Results and discussion

### 3.1 Feature derivation

The PDB IDs, along with the corresponding human proteins and their interacting viral proteins, are listed in Supplementary Table S1. The sequences of these complexes were retrieved from Uniprot. The 18 features were calculated, and all 73 Human-Virus protein complexes were compiled as dataframes (Supplementary Table S1).

The interface residues were labelled using PDBePISA, Intercaat, and a custom PyMOL script, with residues assigned 0 if RSA = 0. Next, a new co-evolution algorithm (Figure 2; Section 2.3.1) generated a non-symmetric M × N matrix. This approach improved detection of co-evolving residues compared to traditional L × L matrices and PSSM-based methods (Afsar et al., 2014; Maheshwari and Brylinski, 2016; Chen and Zhou, 2005; Murakami and Mizuguchi, 2010; Sanchez-Garcia et al., 2019). To further enhance the analysis, structural and environmental features-including RSA, charge, hydropathy, size compatibility, secondary structure, and Miyazawa-Jernigan contact potentials (Miyazawa and Jernigan, 1996)-were incorporated. Finally, ANN training was performed both with and without environmental context to evaluate their impact on predictive accuracy.

### 3.2 Labelling of the dataset

The interface-noninterface labels were highly imbalanced, with substantially more 0s than 1s. An initial downsampling strategy, following the approach used in CoRNeA, was applied by randomly selecting an equal number of 0s to match the number of 1s using NumPy; however, this resulted in the loss of informative negative samples. The dataset (73 CSV files) was split. The data imbalance issue was addressed in the training set. To address this, multiple imbalance-handling methods available in the imblearn package-undersampling, oversampling, SMOTE, combined over-undersampling, and K-means-Tomek links undersampling were evaluated. All performance metrics reported in Table 1 were evaluated on a held-out test dataset that was not subjected to any resampling. Imbalance-handling techniques were applied exclusively to the training data to prevent data leakage and ensure unbiased evaluation. Due to the inherent class imbalance in residue-pair datasets, accuracy alone can be misleading, as models biased toward the majority class may achieve high accuracy despite poor minority class prediction. In contrast, SMOTE enables improved learning of minority class patterns, resulting in balanced performance across precision, recall, and F1-score. The SMOTE oversampling produced the most favourable balance between class representation and model performance and was therefore used in subsequent analyses.

**TABLE 1:** Evaluation statistics for ANN classifiers with 5 data balancing algorithms.

Model	Accuracy	Precision	Recall	F1-score
Undersampling	0.69	0.69	0.69	0.69
Oversampling	0.88	0.88	0.88	0.88
SMOTE (A method of oversampling)	0.98	0.98	0.98	0.98
A combination of oversampling and undersampling	0.84	0.82	0.81	0.82
Ktomelinks (A method of undersampling)	1.0	0.5	0.5	0.5

The imbalance between interface and non-interface residue pairs is an inherent characteristic of protein–protein interaction datasets, as interface regions typically represent a small fraction of the total residue space. To address this, multiple imbalance-handling strategies were evaluated, including undersampling, oversampling, SMOTE, and hybrid approaches. Among these, SMOTE provided the best balance between precision and recall (Table 1).

The dataset exhibited a strong class imbalance prior to sampling (interface:non-interface ≈0.00023:1), which was effectively balanced (∼1:1) after applying SMOTE to the training set (Supplementary Table S2). Despite the increased sample size, SMOTE was applied only to the training data, and no synthetic samples were introduced into validation or test datasets.

### 3.3 Evaluation of environment features in ANN classifier

**FIGURE 4:**
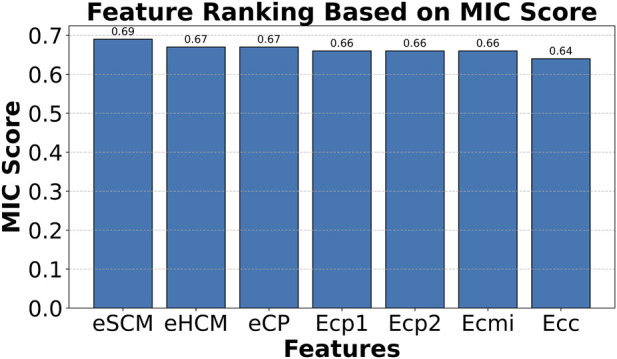
The Top 7 features from 18 features were ranked by mutual information classification scores. The total features include cmi, cc, cp, cp1, cp2, hcm, rsa, scm, ssp, ecc, ecmi, ecp, ecp1, ecp2, ehcm, ersa, escm and essp. The mutual information classification scores of these features are 0.5, 0.05, 0.06, 0.07, 0.06, 0.05, 0.08, 0.07, 0.22, 0.64, 0.66, 0.67, 0.66, 0.66, 0.67, 0.57, 0.69and 0.12, respectively.

**FIGURE 5:**
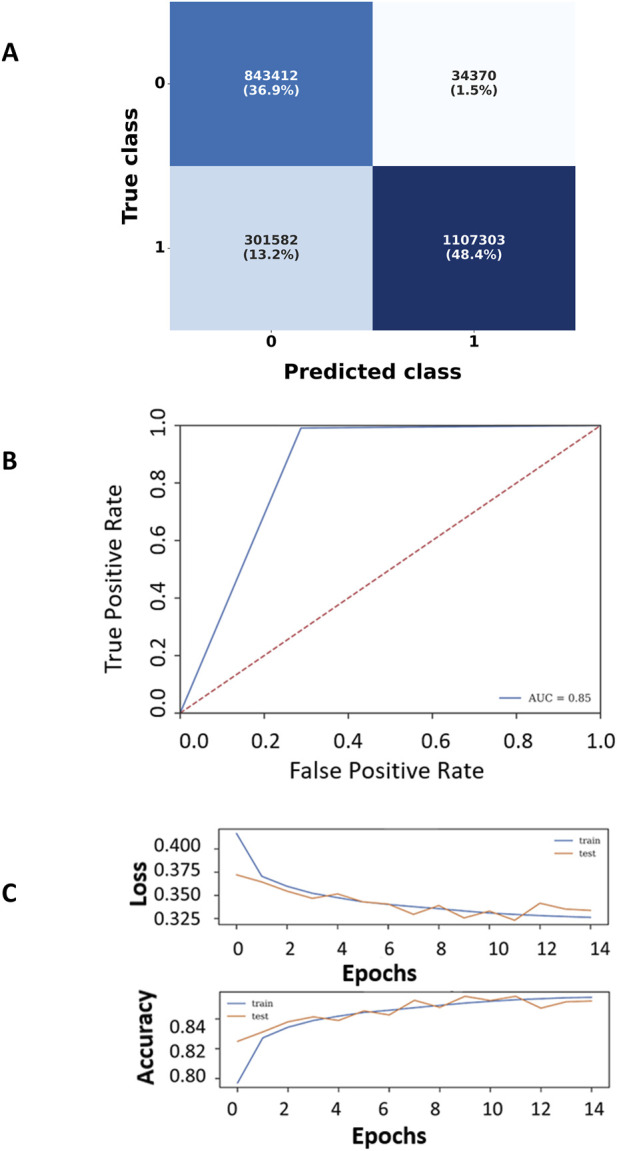
**(A)** Confusion matrix of the ANN model trained with the Top 7 features out of 18 features. **(B)** ROC curve of the model, **(C)** Accuracy and loss plot of the ANN model trained with the Top 7 features out of 18 features.

The feature importance analysis revealed distinct top features for models trained with and without environmental descriptors. With environmental features (18 total), the top seven features were escm, ehcm, ecp, ecp1, ecp2, ecmi, and ecc, whereas cmi, scm, rsa, hcm, cp, cp1, and cp2 ranked highest without them (Figure 4). Performance comparisons (Table 2), supported by confusion matrices, ROC curves, and accuracy-loss plots (Figures 5A–C; Supplementary Figure S2 (file name: Supplementary_figures.pptx; slide 2), 9 features), showed that incorporating environmental features improved overall accuracy from 80% to 85%.

**TABLE 2:** Evaluation statistics for ANN classifiers with and without environmental features.

Model	Accuracy	Precision	Recall	F1-Score
With environment features	0.85	0.86	0.86	0.86
Without environmental features	0.80	0.84	0.79	0.78

The comparative analysis highlights the impact of environmental features and the improved performance of HVIface over CoRNeA. HVIface with environmental features achieves the highest performance, with an accuracy of 0.85 and balanced precision, recall, and F1-score of 0.86, indicating robust and reliable interface prediction. In contrast, removing environmental features leads to a noticeable decline in performance (accuracy: 0.80; F1-score: 0.78), underscoring their importance in capturing interaction context. A similar trend is observed for CoRNeA, where inclusion of environmental features improves performance (F1-score: 0.66 vs. 0.61); however, its overall metrics remain consistently lower than HVIface. Notably, HVIface outperforms CoRNeA across all evaluation metrics in both settings, demonstrating that the proposed enhancements contribute significantly to improved predictive accuracy and better balance between precision and recall.

Notably, the most informative features predominantly correspond to environmentally weighted charge compatibility (ecc, ehcm) and co-evolution–derived contact potentials (ecmi, ecp variants). This trend indicates that viral–host interfaces are strongly governed by electrostatic complementarity within structurally coherent residue neighborhoods rather than by isolated residue contacts. The enrichment of environmental charge compatibility suggests that viral proteins preferentially target pre-organized electrostatic patches on host proteins, consistent with a hijacking strategy that relies on surface complementarity and minimal structural rearrangement.

### 3.4 Type of secondary structures in the interaction hotspots and feature kernel matrix size relationship

The influence of kernel matrix size on classifier performance was evaluated because residue-environment features (ecc, ecmi, ecp, ecp1, ecp2, ehcm, and escm) emerged as the most informative in ANN training. Since residue environments can extend from n-1 to n+1 and from n-3 to n+3, classifiers were trained using features derived from multiple kernel sizes and weights. Each ANN model was assessed across all test datasets to determine whether kernel dimensions or secondary-structure interaction types affected predictive performance. Optimized models were further validated on interacting protein pairs with known crystal structures excluded from training. Among all configurations, the 5 × 5 kernel matrix yielded the most accurate interaction predictions.

### 3.5 Evaluation of ANN trained models

The dataset (73 protein complexes in CSV file) was randomly shuffled and split into 80% training and 20% testing. Regarding dataset size, the 73 complexes may appear limited compared to large-scale intra-species PPI datasets. However, experimentally resolved human–virus protein–protein interaction complexes with residue-level interface annotation are inherently scarce. Our dataset was curated to ensure structural quality, non-redundancy, and biologically validated interface information. Moreover, the total number of residue-pair samples used for training is substantially larger due to combinatorial residue pairing within complexes.

The ANN model employed a *he_uniform* kernel initializer, ReLU activation in all hidden layers, and a sigmoid function in the output layer. Training was performed using the ADAM optimizer (learning rate = 0.001), with *binary_crossentropy* as the loss function and accuracy as the evaluation metric. The model was trained for a maximum of 50 epochs; however, early stopping was applied based on validation performance, resulting in convergence at 14 epochs. The training and validation accuracy and loss curves are shown in Figure 5C. The model performance was subsequently assessed on the test set. The optimized ANN trained on the top 18 environmental features was used for interface prediction.

To evaluate the robustness of HVIface, we performed 10-fold cross-validation using a complex-level splitting strategy. The model achieved a mean accuracy of 0.853 ± 0.035 and a mean ROC-AUC of 0.705 ± 0.061, indicating stable and reliable performance across folds (Table 3).

**TABLE 3:** Performance of HVIface using 10-fold cross-validation at the complex level.

Fold	Accuracy	ROC-AUC
1	0.853457272	0.638919874
2	0.848245919	0.690732477
3	0.827049315	0.664137109
4	0.843927264	0.702347316
5	0.814273596	0.648177449
6	0.839121878	0.706119751
7	0.859039307	0.848669674
8	0.936605215	0.716282257
9	0.827613056	0.678351314
10	0.885629177	0.756393789
Mean ± SD	0.853 ± 0.035	0.705 ± 0.061

To further assess generalization across viral species, we conducted Leave-One-Family-Out (LOFO) validation. The model maintained high predictive accuracy across different viral families, ranging from 0.836 to 0.882, demonstrating strong classification capability (Table 4). However, ROC-AUC values showed variability across families, with lower performance observed for SARS-CoV-2 (AUC = 0.582) compared to Influenza (AUC = 0.719) and HIV (AUC = 0.688).

**TABLE 4:** Leave-One-Family-Out (LOFO) validation of HVIface across different viral families.

Viral family	Test complexes	Accuracy	ROC-AUC
HIV	14	0.88230592	0.687835339
Influenza	11	0.858278692	0.718903753
Other	21	0.862340331	0.620595335
SARS-CoV-2	27	0.835623443	0.582488274

This variation likely reflects differences in structural diversity and interaction patterns among viral families, highlighting the inherent complexity of host–virus interface prediction. Nevertheless, the model consistently outperformed random classification, indicating meaningful predictive capability even in cross-family scenarios.

### 3.6 Model evaluation report on 16 test datasets

Table 5 summarises the confusion matrix statistics for all test cases before and after network analysis, and Table 5 presents the evaluation results for the 16 test cases. Supplementary Figures (Supplementary Figures.pptx, slides 3–18) provide the ROC curves, confusion matrices, and interaction poses in both complex and open-book views for all 16 test cases. Detailed prediction outputs and evaluation metrics for each of the 16 test cases are provided in Supplementary Material.doc (pp. 3–33).

**TABLE 5:** Confusion matrix statistics for all test cases before and after network analysis.

PDB ID	Method	True negative	False positive	False negative	True positive
7UC5	ANN	30,121	29,690	9	12
	After network and convolution	36,676	23,128	10	18
7T0O	ANN	142,822	97,660	7	11
	After network and convolution	149,977	90,502	7	14
7B7N	ANN	90,475	55,932	5	3
	After network and convolution	92,474	53,932	4	5
6I2M	ANN	55,684	44,854	26	20
	After network and convolution	61,203	39,228	35	26
5IRO	ANN	17,510	12,135	27	28
	After network and convolution	25,858	3787	2	53
2JDQ	ANN	23,278	14,004	33	35
	After network and convolution	34,410	2872	8	60
3TO2	ANN	34,747	25,979	20	29
	After network and convolution	59,678	1048	2	47
6BVV	ANN	11,035	7692	43	49
	After network and convolution	17,548	1179	8	84
7KDT	ANN	29,738	18,658	50	54
	After network and convolution	46,377	2019	10	94
6E5U	ANN	48,761	28,951	7	5
	After network and convolution	75,116	2596	2	10
6JO8	ANN	70,992	45,200	5	11
	After network and convolution	102,062	14,130	0	16
7M4R	ANN	17,348	12,095	15	17
	After network and convolution	25,147	4296	5	27
7P9W	ANN	21,602	11,726	37	24
	After network and convolution	29,165	4163	20	41
4U0C	ANN	27,212	16,427	13	7
	After network and convolution	42,523	1116	2	18
4U0A	ANN	38,728	22,457	15	15
	After network and convolution	57,362	3823	3	27
1Q94	ANN	44,956	30,647	9	13
	After network and convolution	65,605	9998	5	17

The dataset was split at the complex level into training and test sets, with 80% of complexes used for training and 20% (16 complexes) used for testing. The test set comprises a diverse range of viral families, including HIV, influenza, SARS-CoV-2, human herpesvirus, Vaccinia virus, Adenovirus, Nipah virus, Chikungunya virus, and Epstein–Barr virus (Table 6), ensuring that evaluation is not biased toward a specific viral class.

Model performance across individual test complexes (Table 6) shows variability, with accuracy ranging from ∼61% to 98%, reflecting the inherent structural and interaction diversity among host–virus systems.

**TABLE 6:** Evaluation metrics on 16 test datasets.

PDB ids	Virus	Model accuracy	Precision	Recall	F1-score
7UC5	Influenza virus	61%	0.99	0.61	0.76
7T0O	HIV	62%	0.99	0.62	0.76
7B7N	Human herpes virus	63%	0.99	0.63	0.77
6I2M	Vaccinia virus	61%	0.99	0.60	0.75
5IRO	Adenovirus	87%	0.99	0.87	0.93
2JDQ	Influenza virus	92%	0.99	0.92	0.95
3TO2	SARS-CoV-2	98%	0.99	0.98	0.99
6BVV	Nipah virus	94%	0.99	0.94	0.96
7KDT	SARS-CoV-2	96%	0.99	0.95	0.97
6E5U	Influenza virus	97%	0.99	0.96	0.98
6JO8	Chikungunya	88%	0.99	0.87	0.93
7M4R	SARS-CoV-2	85%	0.99	0.85	0.92
7P9W	Epstein-barr virus	87%	0.99	0.87	0.93
4U0A	HIV	94%	0.99	0.93	0.96
4U0C	HIV	97%	0.99	0.97	0.98
1Q94	HIV	87%	0.99	0.86	0.92

Although the dataset size is limited, this reflects the scarcity of experimentally resolved host–virus complexes. To address potential overfitting and improve robustness, 10-fold cross-validation and Leave-One-Family-Out (LOFO) validation were performed. Additionally, dropout regularization and early stopping were applied during model training.

**FIGURE 6:**
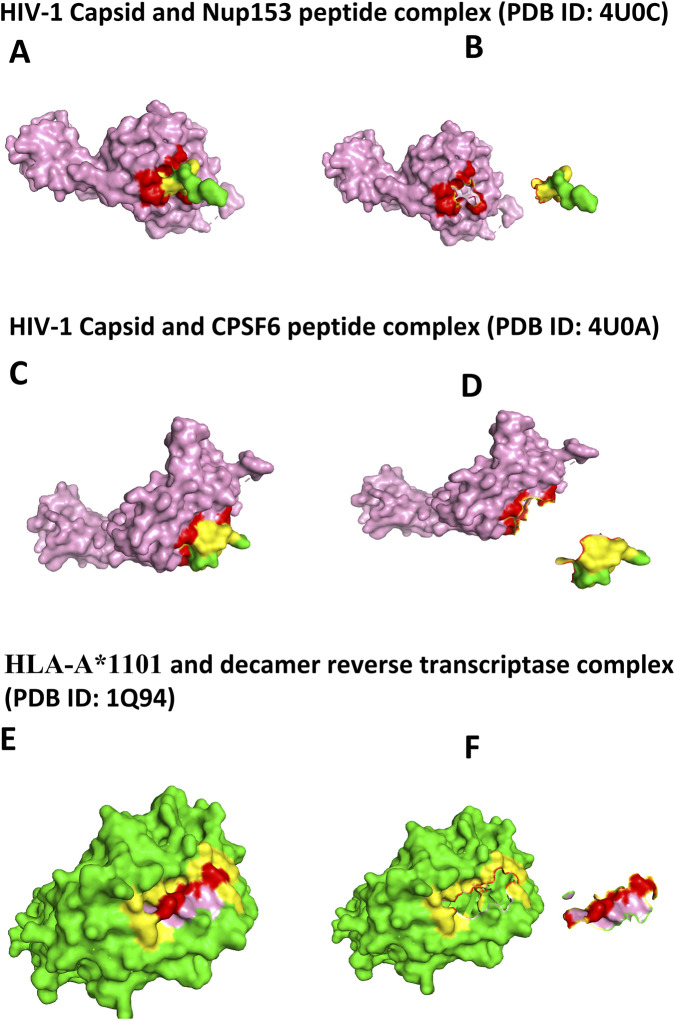
**(A)** Nup153-Capsid complex: Nup153 is shown in green with interface residues in yellow and the capsid protein is shown in pink with interface residues in red. **(B)** Interfaces predicted by PISA but not by HVIface for the Nup153-Capsid complex: Nup153 interface residues are highlighted in cyan and capsid interface residues are shown in forest green. **(C)** CPSF6-Capsid complex: CPSF6 is shown in green with interface residues in yellow and the capsid protein is shown in pink with interface residues in red. **(D)** Interfaces predicted by PISA but not by HVIface for the CPSF6-Capsid complex: CPSF6 interface residues are shown in cyan and capsid interface residues are displayed in forest green. **(E)** HLA-A*1101-Reverse Transcriptase complex: HLA-A1101 is shown in green with interface residues in yellow and reverse transcriptase is shown in pink with interface residues in red. **(F)** Interfaces predicted by PISA but not by HVIface for the HLA-A1101-Reverse Transcriptase complex: HLA-A*1101 interface residues are shown in cyan and reverse transcriptase interface residues are shown in forest green.

For the HIV-1 capsid-Nup153 complex (PDB ID 4U0C), the trained ANN model achieved 93% interface prediction accuracy (Tables 7, 8; Figures 6A,B). In the HIV-1 capsid-CPSF6 complex (PDB ID 4U0A), the model reached an accuracy of 84% (Tables 7, 8; Figures 6C,D). For the HLA-A1101-HIV-1 reverse transcriptase epitope complex (PDB ID 1Q94), the model accurately identified interface residues with 99% accuracy (Tables 7, 8; Figures 6E,F).

**TABLE 7:** Evaluation metrics of the model in the prediction of interfaces in the three PDB complexes.

PDB IDs	Model accuracy	Precision	Recall	F1-score
4U0C	93%	100%	93%	96%
4U0A	84%	100%	84%	91%
1Q94	99%	100%	99%	99%

**TABLE 8:** The interacting residues predicted by the model in the three PDB complexes.

PDB ID 4U0C: Nup153-capsid protein interface residues	PDB ID 4U0A: CPSF6-capsid protein interface residues	PDB ID 1Q94: HLA-A*1101-reverse transcriptase protein interface residues
F1415-T54	V314-G101	E63-A746
F1415-N57	L315-Q67	E63-I747
T1416-Q63	F316-Q67	Y99-F748
T1416-Q67	P317-Q67	Q70-S750
F1417-N53	G322-G106	Y159-A746
F1417-K70	P325-T107	W147-T753
G1418-N53	V314-N74	K146-T753
G1418-A105	F321-N57	T143-K754
G1418-G106	L315-N74	
V1414-R173		
T1416-R173		
S1412-Q176		
S1412-A177		
F1417-L56		

The accuracy of HVIface shows a clear dependence on sequence conservation. Complexes involving highly conserved viral proteins, such as HIV capsid and reverse transcriptase (e.g., PDB IDs 4U0C and 1Q94), exhibit high prediction accuracy (93%–99%), reflecting strong co-evolutionary signals derived from deep MSAs. In contrast, systems involving conformationally flexible and less conserved viral proteins, such as gp140 in the CD4–gp140 complex (PDB ID 7T0O), show reduced accuracy (∼62%), consistent with weaker sequence conservation and noisier co-evolutionary features. This trend highlights an inherent limitation of sequence-based interface prediction methods rather than a system-specific failure of HVIface.

### 3.7 Evaluation of HVIface on case studies

#### 3.7.1 LASP1-NS5A

**FIGURE 7:**
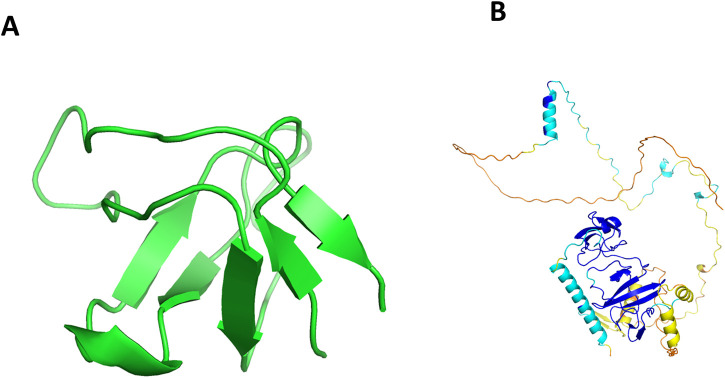
**(A)** Human SH3 domain of protein LASP1 (PDB ID 3I35). **(B)** The alphafold structure of NS5A.

LASP1 is a human actin-regulatory protein (Pollitt et al., 2020), and its phosphorylation status modulates actin-associated ion transport in several epithelial cell types (Chew et al., 2002). The HCV non-structural protein NS5A, which exhibits cysteine-type endopeptidase activity, has been reported to interact with LASP1 based on luciferase reporter and co-localization assays (Pliss et al., 2016); however, no structural information is available for the LASP1-NS5A complex. Using HVIface, we determined that the C-terminal SH3 domain of LASP1 (residues 202–261; PDB ID 3I35; Figure 7A) constitutes the primary interface predicted to interact with NS5A Domain I (Choi et al., 2020). To complement the sequence-based predictions, we also performed docking using the LASP1 crystal structure and the AlphaFold3.0 model of NS5A (Figure 7B).

**FIGURE 8:**
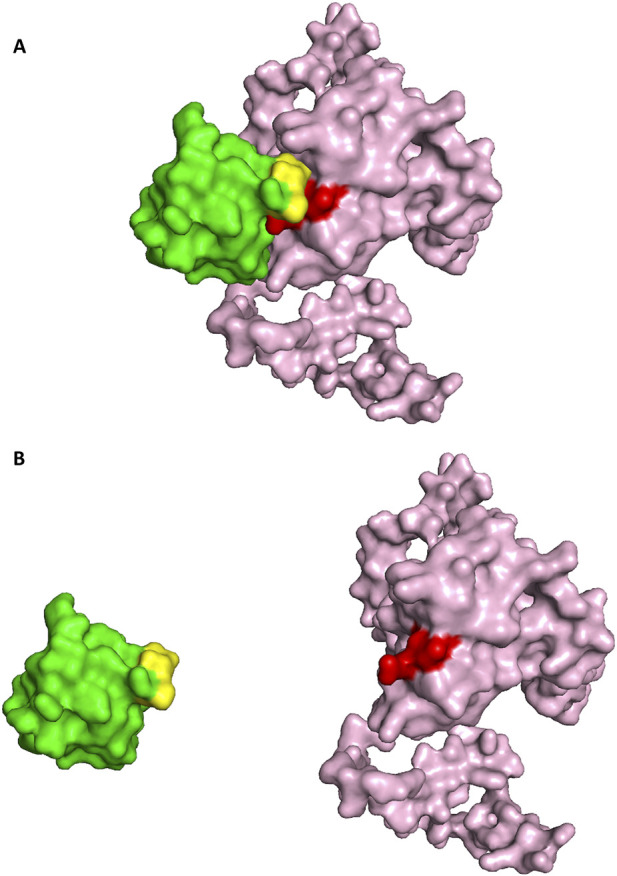
**(A)** Docked pose Human LASP1-NS5A (LASP1-Green color: interface(yellow color)-NS5A-pink color: interface(red color)). **(B)** Open book conformation of the docked.

The top 10% ANN-based interface predictions (Table 9) highlighted a consistent cluster of LASP1 residues-A216, D217, and E218-engaging NS5A residues 75–77. These ANN-identified residue pairs showed high interaction scores (Lu et al., 2025; Guo et al., 2008; Romero-Molina et al., 2019; Ma et al., 2020; Ahmed et al., 2018; You et al., 2015) and short predicted spatial distances (1.4–4.4 Å), strongly suggesting a compact, well-defined interaction patch on the SH3 domain. Notably, residue E218 exhibited multiple high-confidence contacts (E218-R75, E218-I76, E218-V77), indicating its central role as an anchoring residue within the interface. Importantly, these ANN-derived predictions aligned closely with the highest-ranked docking pose (score −126.97), where the same residues formed the core of the binding interface (Figures 8A,B). The convergence of high neural-network confidence, favorable geometric distances, and structural docking robustly demonstrates HVIface’s ability to delineate the LASP1-NS5A interaction surface.

**TABLE 9:** The prediction of the interaction between LASP1 and NS5A.

Human LASP1	HCV NS5A: pLDDT score	Prediction score	Distance (Å)
216A	76I:95.95	11	3.0
216A	77V:97.46	14	4.4
217D	75R:84.40	10	3.3
218E	75R:84.40	11	1.4
218E	76I:95.95	15	4.2
218E	77V:97.46	13	4.0

The interaction analysis between human LASP1 and HCV NS5A reveals that the predicted interface residues are associated with moderate-to-high structural confidence, as indicated by pLDDT scores ranging from 84.40 to 97.46 for NS5A (Table 9). Notably, residues such as 76I and 77V exhibit very high confidence (pLDDT >95), supporting the reliability of the predicted interactions. The identified residue pairs display favorable spatial proximity, with inter-residue distances ranging from 1.4 Å to 4.4 Å, consistent with plausible molecular contacts. Additionally, the corresponding prediction scores (ranging from 10 to 15) further reinforce the strength of these interactions. Overall, the convergence of high pLDDT values, strong prediction scores, and close spatial distances supports the robustness of the LASP1–NS5A interface predicted by the HVIface model.

#### 3.7.2 Nup85-PB1

**FIGURE 9:**
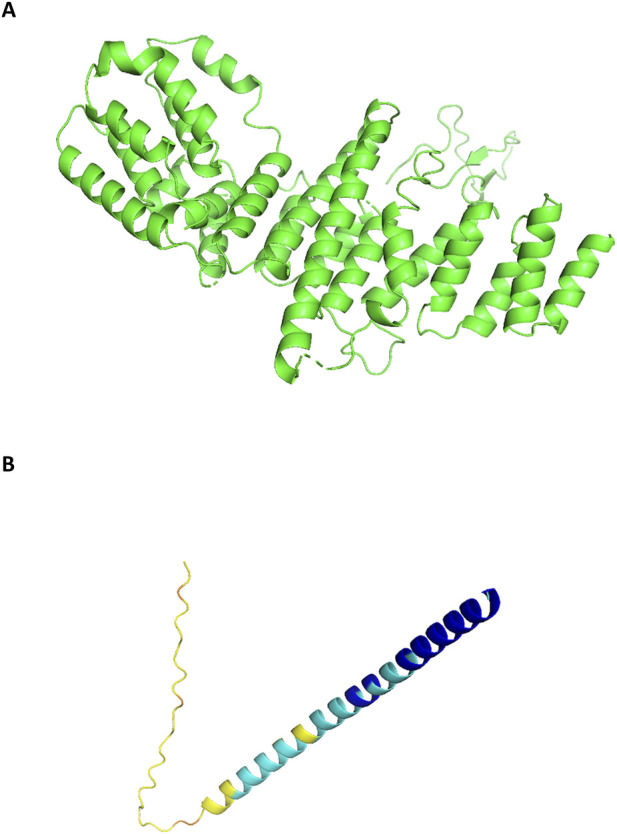
**(A)** Human Nup85 (PDB ID 5A9Q). **(B)** The alphafold structure of PB1.

Nup85 is a Human Nucleoporin involved in mRNA export (Ling et al., 2022). Although Influenza PB1 has been reported to associate with Nup85 in an RNA-dependent manner, no structural information is available for this complex. The human Nup85 structure (656 residues) is available in the PDB (ID: 5A9Q), whereas PB1 (87 residues) was modeled using AlphaFold. These structures were used for molecular docking (Figures 9A,B), and the top 10% HVIface predictions (Table 10) were evaluated to identify putative interface regions.The interaction analysis between human Nup85 and Influenza virus PB1 indicates that the predicted interface residues are supported by moderate structural confidence, with PB1 pLDDT scores ranging from 62.14 to 82.36. Residues such as 54Q, 57Y, and 55E fall within moderate-to-high confidence regions, suggesting reasonable reliability of the modeled interface. The predicted residue pairs exhibit close spatial proximity, with inter-residue distances between 2.3 Å and 4.2 Å, consistent with potential molecular contacts. Although the prediction scores are relatively modest (ranging from 3 to 6), the agreement between structural proximity and acceptable pLDDT values supports the plausibility of the Nup85–PB1 interaction interface identified by the model.

**TABLE 10:** The prediction of the interaction between Nup85 and PB1.

Human Nup85	Influenza virus PB1	Prediction score	Distance (Å)
100R	46T:62.14	6	2.3
106R	54Q:75.83	6	4.2
107S	54Q:75.83	6	2.6
114E	57Y:79.35	3	2.4
115E	57Y:79.35	3	2.7
158P	55E:82.36	5	4.2

**FIGURE 10:**
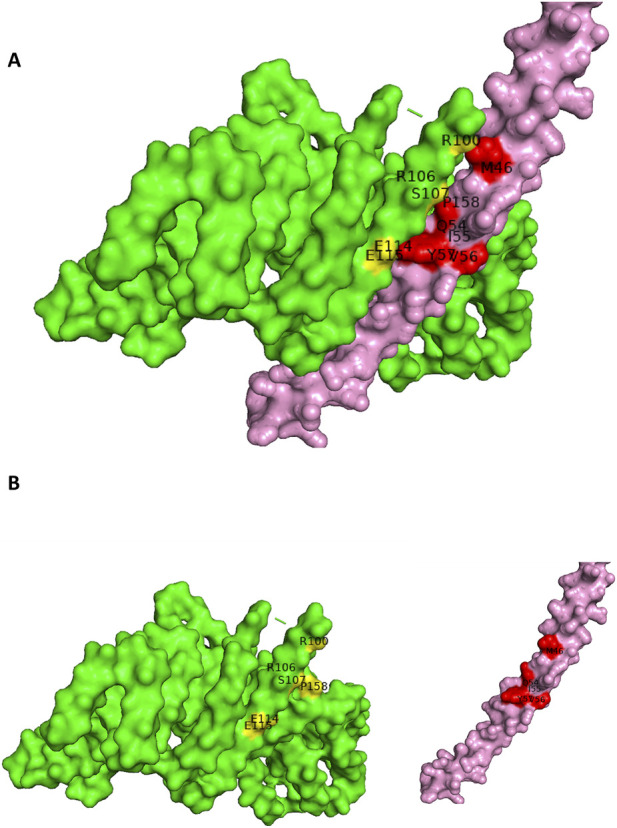
**(A)** Docked pose Human Nup85-PB1 (Nup85-Green color: interface(yellow color)-PB1-pink color:interface(red color)). **(B)** Open book conformation of the docked.

The ANN model predicted a focused interface cluster on Nup85 involving residues R100, R106, S107, E114, and E115, which formed contacts with PB1 residues T46, Q54, and Y57. These residue pairs showed moderate-to-high interaction scores (Mallah et al., 2021; Koonin et al., 2022; Li et al., 2022; Lin et al., 2017) and short predicted spatial distances (2.3–4.2 Å), indicating that the network consistently localized the interaction to a compact surface region on Nup85. Notably, PB1 residue Q54 appeared repeatedly among the highest-confidence contacts (with R106 and S107), suggesting its central role in the interaction interface. These ANN-derived predictions were in strong agreement with the highest-ranked docking pose (−284.14), in which the same Nup85 surface patch formed the core of the binding interface (Figures 10A,B). The convergence of predicted residue proximity, ANN confidence scores, and structural docking reinforces the reliability of HVIface in identifying the Nup85-PB1 interaction interface.

The scores reported in Tables 8, 9 represent convolutional scores obtained after applying a 5 × 5 kernel over the feature matrices to capture local residue environment information. These scores reflect the interaction strength between residue pairs, with higher values indicating stronger interaction propensity. As a general guideline, residue pairs with convolutional scores greater than 5 are considered to represent reliable and high-confidence interactions.

#### 3.7.3 HIV-1 integrase and human Nup62

**FIGURE 11:**
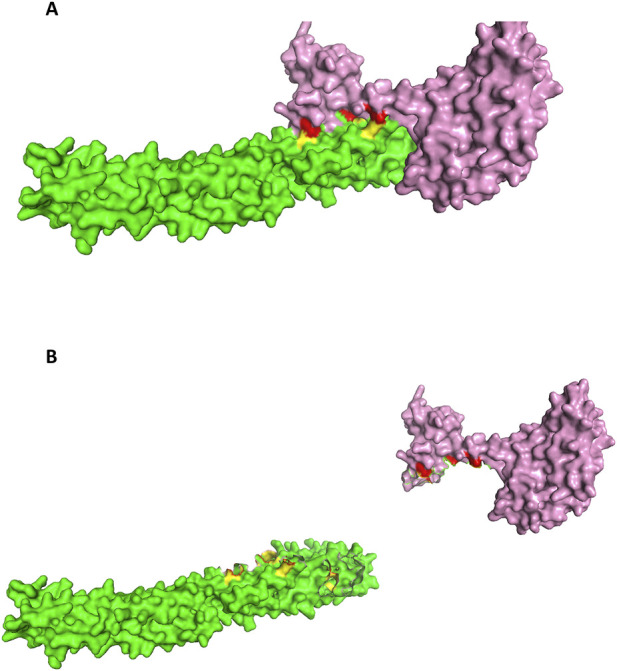
**(A)** The docked pose of Dimeric Nup62(Nup62-Green color: interface(yellow color))-Integrase (pink color: interface(red color)). **(B)** Open book conformation of the docked Dimeric Nup62-HIV Integrase.

The Nup62 is a central nucleoporin essential for mitotic progression and Nuclear Pore Complex (NPC) organization, consisting of a structured coiled-coil region and an intrinsically flexible FG-repeat domain (Madheshiya et al., 2022). Its role in assembling diverse Nup62-containing heterotrimers is well established. HIV-1 Integrase has been shown to associate with human Nup62, with pull-down assays demonstrating that the C-terminal fragment Nup62(328–522) binds more strongly than full-length Nup62(1–522) (Ao et al., 2012). Although no structure of the Nup62-Integrase complex exists, structural information is available for the Nup62 dimeric C-terminal domain (PDB ID: 5IJN) (Dew et al., 2017) and for the catalytic core domain of Integrase (PDB ID: 6NUJ). Docking of dimeric Nup62 with integrase produced a favorable score of −244.84, and the resulting interface (Figures 11A,B) showed close correspondence to the top 10% ANN-predicted contacts (Table 11). The interaction analysis between human Nup62 and HIV-1 Integrase reveals that the predicted interface residues span a wide range of structural confidence, with pLDDT scores varying from low (26.62) to moderate (65.92). While certain residues, such as 215K (pLDDT ∼65.92), fall within relatively reliable regions, several interacting residues are located in lower-confidence regions, suggesting potential structural flexibility or disorder in parts of the viral protein. Despite this variability, the predicted residue pairs exhibit close spatial proximity, with inter-residue distances ranging from 1.2 Å to 4.6 Å, consistent with feasible molecular contacts. The relatively high prediction scores (Guo et al., 2008; Romero-Molina et al., 2019; Ma et al., 2020) further support the potential significance of these interactions. Overall, while the interaction interface is supported by favorable geometric and prediction metrics, the presence of lower pLDDT regions suggests that these interactions should be interpreted with caution and may benefit from further experimental validation.

**TABLE 11:** The prediction of the interaction between Dimeric Nup62 and Integrase.

Human Nup62	HIV integrase	Prediction score	Distance (Å)
384L:B	274Q: 37.02	13	1.2
490Q:B	222N: 60.99	12	1.6
339K: A	55D: 39.31	11	2.5
489D:B	272G: 44.50	12	4.6
456E:B	215K: 65.92	12	2.0
378E:B	285Q: 26.62	12	3.9

The ANN model identified six high-confidence interface pairs for the dimeric complex, characterized by high interaction scores (Guo et al., 2008; Romero-Molina et al., 2019; Ma et al., 2020) and short predicted distances (1.2–4.6 Å). These included Nup62 residues L384, Q490, D489, E456, and E378, making contacts with Integrase residues Q274, N222, G272, K215, and Q285, each repeatedly highlighted by the ANN as a central determinant of the interaction. In particular, interactions involving Q490:B-N222 and L384:B-Q274 were among the strongest predictions, suggesting that the dimeric Nup62 C-terminal helical bundle provides a structurally stable interface for integrase engagement. This clustering of high-confidence ANN predictions on the dimeric Nup62 surface closely aligned with the docking-derived interface, underscoring the robustness of HVIface in identifying biologically relevant interaction determinants.

**FIGURE 12:**
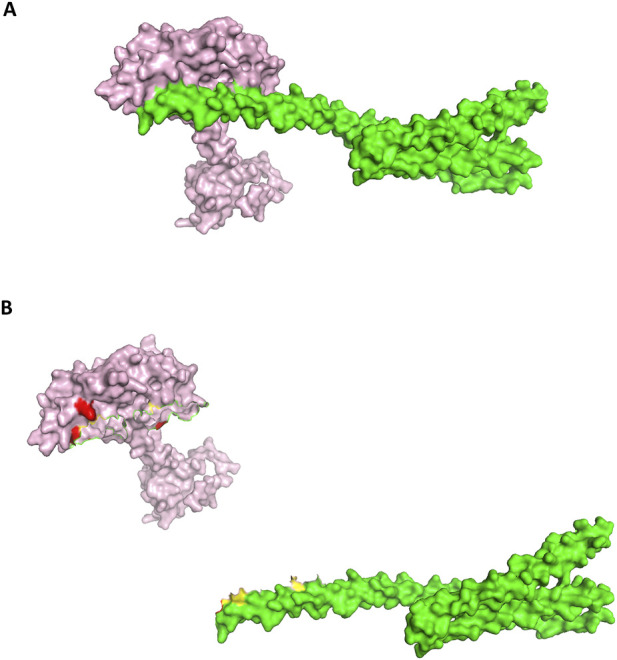
**(A)** Docked pose Human monomeric Nup62-HIV Integrase (Nup62-Green color: interface(yellow color))-Integrase (pink color: interface(red color)). **(B)** Open book conformation of the docked monomeric Nup62-HIV Integrase.

In contrast, the monomeric Nup62 docking pose (score −268.24) produced interface contacts (Table 12; Figures 12A,B) that were not supported by ANN predictions. Although several residue pairs showed close geometric proximity (e.g., Y332-D25 at 3.86 Å, Q350-G106 at 3.83 Å), the ANN model did not assign interaction scores to any of these contacts, indicating low sequence-based confidence for monomeric recognition. This divergence between docking geometry and ANN predictions suggests that monomeric Nup62 may form transient contacts with integrase, whereas the dimer provides the physiologically relevant binding configuration.

**TABLE 12:** The prediction of the interaction between Monomeric Nup62 and Integrase.

Human Nup62	HIV Integrase: pLDDT score	Distance (Å)	Prediction score
332Y	25D:79.08	3.86	Not predicted by the model
350Q	106G: 92.19	3.83	
330M	26F:79.85	3.85	
332Y	186K: 80.86	2.24	
336Q	14K: 82.08	3.84	

**FIGURE 13:**
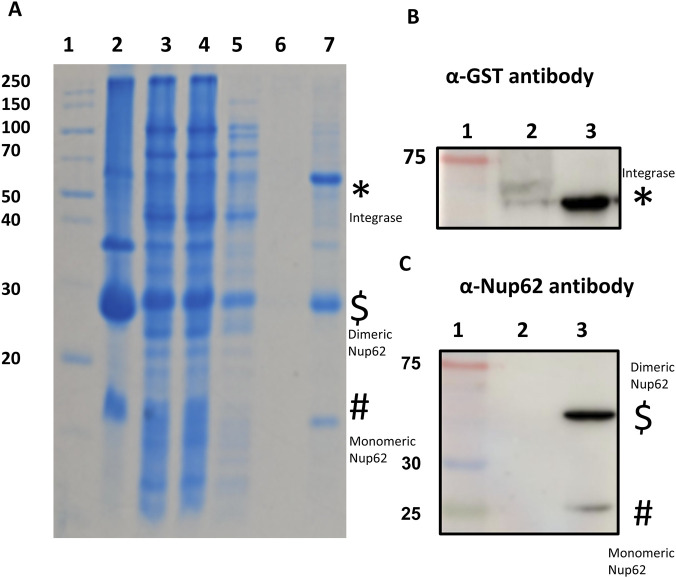
**(A)** GST Affinity pull down HIV Integrase-Nup62; 1- Marker, 2- Pellet, 3- Supernatant, 4- Flowthrough, 5- First wash, 6- Last wash, 7- Elution 1; Here the monomeric Nup62 (26 kDa) is represented by #; the dimeric Nup62 is represented by $; the integrase is represented by * **(B)** 1- Marker, 2- Flowthrough, 3- Pull down. Western blot of GST-tagged HIV Integrase (58 kDa) probed with α-GST antibody represented by * **(C)** 1- Marker, 2-Flowthrough, 3- Pull down. Western blot of His-tagged Nup62(Monomeric: 26 kDa and Dimeric: 52 kDa) probed with α-His antibody represented by # and $, respectively.

These computational observations are consistent with experimental pull-down assays (Figures 13A–C), which exhibited that both monomeric (26 kDa) and dimeric (52 kDa) Nup62 can engage HIV-1 Integrase (58 kDa), but the dimer shows markedly higher binding affinity. Together, the convergence of ANN high-confidence predictions, favorable docking energetics, and biochemical validation strongly supports a model in which HIV-1 Integrase preferentially recognizes the dimeric Nup62 C-terminal domain, aligning with its broader dependence on nucleoporins such as Nup153 during nuclear import of the viral pre-integration complex (Woodward et al., 2009).

To further evaluate the structural reliability of the modelled host–virus complexes, we assessed the AlphaFold-predicted structures using per-residue confidence scores (pLDDT). The majority of residues involved in the predicted interaction interfaces correspond to moderate-to-high confidence regions, as indicated by pLDDT-based color mapping (ranging from yellow to blue). For visualization, pLDDT values were mapped onto the structures using a continuous color spectrum (red–yellow–green–cyan–blue; minimum = 50, maximum = 90; pymol command: spectrum b, red_yellow_green_cyan_blue, minimum = 50, maximum = 90), allowing clear interpretation of structural confidence across the docked complexes.

Notably, the interface residues identified by HVIface consistently localized within structurally well-defined regions of the proteins. In addition, the docked complexes generated using HDOCK showed strong agreement with the HVIface-predicted interface residues across all case studies, including LASP1–NS5A, Nup85–PB1, and Nup62–Integrase systems. The convergence of sequence-based predictions, docking-derived geometries, and structural confidence metrics further reinforces the reliability of the identified interaction interfaces.

For transparency and reproducibility, the AlphaFold-predicted structures, docked complexes, and corresponding pLDDT annotations have been made publicly available in the validation repository (https://github.com/krishnakantgupta-ai/HVIface_MMAD/tree/main/Validation).

Beyond structural validation, the analyzed complexes reveal recurring physicochemical trends. In multiple cases, viral proteins utilize clusters of charged and polar residues to engage solvent-exposed acidic or hydrophobic host (Human) surface patches, forming compact interaction hotspots. Rather than extensive hydrophobic burial, the interfaces appear dominated by electrostatic complementarity reinforced by localized environmental coherence. This pattern is consistent across unrelated virus families, suggesting that viral hijacking commonly exploits pre-organized host surface features through electrostatic anchoring and minimalistic binding motifs. Such compact, hotspot-driven architectures may reflect evolutionary constraints on viral genome size while preserving binding specificity.

### 3.8 Comparison with other tools

There are many binary-based tools available for protein-protein interaction prediction, including DeepViral (Liu-Wei et al., 2021), SPPS (Liu et al., 2012), TRI_TOOL (Perovic et al., 2017), MP-VHPPI (Asim et al., 2022), and LR_PPI (Pan et al., 2010). PeSTO and PIPENN predict interfacial residues, with PeSTO being structure-based and PIPENN sequence-based. The primary HVIface model was trained using a 10 Å distance cutoff to define interface residues, enabling the capture of both direct contacts and proximal residues contributing to the interaction environment. However, for comparative evaluation with existing methods such as PIPENN and PeSTo, which employ a 5 Å cutoff, a separate model was constructed using this more stringent definition. This ensured a fair and consistent benchmarking framework while preserving the broader interaction context in the primary model. HVIface was compared with these tools (Tables 13, 14) and demonstrated higher accuracy than PIPENN but lower than PeSTO (Table 13). However, across 16 datasets, both HVIface and PIPENN outperformed PeSTO (Table 14), with HVIface performing slightly better than PIPENN. HVIface was trained using co-evolution scores, charge, size, hydropathy compatibility, contact potentials (original, exposed, buried), residue solvent accessibility, and secondary structure. Unique features of HVIface include charge, size, and contact potentials, along with the novel incorporation of environmental characteristics of interacting residue pairs.

**TABLE 13:** Comparison of HVIface with PeSTo and PIPENN.

Tool name	Accuracy	Precision	MCC
PeSTo	89%	69%	0.67
PIPENN	82%	24%	0.192
HVIface	85%	86%	0.87

**TABLE 14:** Comparison of predictions from HVIface with PeSTO (geometry deep learning) and PIPENN (DNN-NET_BioDL_P).

Test dataset	Method	Expected No of residues (contacts) within 5 Å	Number of true positives with probability more than 0.5	Number of false positives with probability more than 0.5
7UC5	PeSTO	22	0	45
	PIPENN		12	23,130
	HVIface		14	23,128
7T0O	PeSTO	17	0	30
	PIPENN		8	90,506
	HVIface		11	90,502
7B7N	PeSTO	4	0	49
	PIPENN		2	53,931
	HVIface		2	53,932
6I2M	PeSTO	52	6	60
	PIPENN		20	39,232
	HVIface		22	39,228
5IR0	PeSTO	47	4	44
	PIPENN		33	3794
	HVIface		41	3787
2JDQ	PeSTO	60	8	66
	PIPENN		51	2871
	HVIface		52	2872
3TO2	PeSTO	30	2	32
	PIPENN		17	1054
	HVIface		19	1048
6BVV	PeSTO	66	5	87
	PIPENN		43	1180
	HVIface		49	1179
7KDT	PeSTO	72	0	58
	PIPENN		30	2026
	HVIface		45	2019
6E5U	PeSTO	6	0	12
	PIPENN		4	2600
	HVIface		4	2596
6JO8	PeSTO	12	0	8
	PIPENN		6	14,132
	HVIface		7	14,130
7M4R	PeSTO	20	0	28
	PIPENN		10	4310
	HVIface		18	4296
7P9W	PeSTO	42	3	53
	PIPENN		25	4181
	HVIface		25	4163
4U0C	PeSTO	12	0	14
	PIPENN		5	16,432
	HVIface		8	16,427
4U0A	PeSTO	14	5	26
	PIPENN		7	3836
	HVIface		12	3823
1Q94	PeSTO	16	0	13
	PIPENN		11	10,005
	HVIface		11	9998

HVIface is, to our knowledge, the first method developed to systematically study human–virus protein–protein interaction interfaces. Nevertheless, the method relies on high-quality sequence or structural information, and prediction accuracy may decrease for proteins exhibiting low sequence conservation or lacking complete structural data.

### 3.9 Global interface chemistry trends

To evaluate whether HVIface captures generalizable interface determinants, we analyzed residue-type enrichment across the 73 human–virus complexes. Viral interface residues exhibit a significant enrichment of charged and polar amino acids relative to non-interface surface residues, whereas hydrophobic residues are more frequently concentrated on the host side of the interface. This compositional asymmetry indicates a recurring recognition pattern in which viral proteins preferentially utilize electrostatic complementarity to engage structurally stable or hydrophobic host surface patches.

Furthermore, the consistent prominence of co-evolutionary features and environmentally weighted charge compatibility across multiple virus families suggests that human–virus interfaces are governed by conserved physiochemical constraints rather than stochastic residue pairing. Viral proteins tend to target solvent-accessible, electrostatically favorable host residues, forming compact interaction clusters that resemble hotspot-centered binding architectures.

Collectively, these observations indicate that human–virus interfaces are characterized by enriched electrostatic complementarity, local environmental residue coherence, and clustered binding hotspots. Such recurring physicochemical patterns support a mechanistic model in which viral hijacking relies on electrostatic anchoring and localized interface optimization, enabling efficient host engagement without extensive hydrophobic burial or large-scale structural mimicry.

## 4 Conclusion

HVIface provides a dedicated framework for residue-level prediction of human–virus protein–protein interfaces, enabling systematic identification of interacting residue pairs directly from sequence-derived features. By integrating physicochemical compatibility, contact potentials, and co-evolutionary signals, the pipeline achieves robust predictive performance while offering insight into the chemical determinants underlying viral–host recognition. The ability to predict interface residues as paired entities facilitates the delineation of minimal interaction regions within heterodimers and can guide biochemical reconstitution experiments. When experimental or homology-based 3D structures are available, HVIface probability maps can further prioritize interface regions for protein–protein docking and structural refinement.

Beyond prediction, our analysis suggests that human–virus interfaces are characterized by electrostatic complementarity and compact hotspot clustering, providing a mechanistic perspective on viral hijacking strategies.

While HVIface demonstrates promising predictive performance, it should be noted that the current study represents a proof-of-concept due to the limited size and diversity of the available dataset. Consequently, the results should be interpreted with caution, and further validation on larger and more diverse datasets is necessary. Users are encouraged to complement HVIface predictions with additional structural or experimental analyses to ensure robust biological interpretation.

Future developments will incorporate dynamic conformational effects and context-dependent interactions to better capture transient and allosteric interface behavior. Expansion of the training dataset to include larger and more diverse human–virus complexes will further improve generalizability, particularly for emerging and novel viral strains. The source code and trained models are publicly available at https://github.com/krishnakantgupta-ai/HVIface_MMAD.

## Data Availability

The original contributions presented in the study are included in the article/Supplementary Material, further inquiries can be directed to the corresponding authors.
