# Supplementary material for: HVIface: sequence-based deep learning for decoding human-virus protein-protein interfaces
Source: Front Bioinform. 2026 May 8;6:1813796. doi: 10.3389/fbinf.2026.1813796 (PMC13194012; doi:10.3389/fbinf.2026.1813796)
Supplement: Supplementary file 1 [file Table1.docx]

#ANN model architecture:

#define model

Import keras

model=Sequential()

model.add(Dense(50,input_dim=18,activation=’relu’,kernel_initializer=’he_uniform’)

keras.layers.Dropout( 0.3, noise_shape=None,seed=None)

model.add(Dense(40, activation=’relu’))

keras.layers.Dropout( 0.4, noise_shape=None,seed=None)

model.add(Dense(30, activation=’relu’))

keras.layers.Dropout( 0.6, noise_shape=None,seed=None)

model.add(Dense(20, activation=’relu’))

keras.layers.Dropout( 0.7, noise_shape=None,seed=None)

model.add(Dense(10, activation=’sigmoid’))

keras.layers.Dropout( 0.8, noise_shape=None,seed=None)

model.add(Dense(1, activation=’sigmoid’))

#model compile

opt=keras.optimizers.Adam(learning_rate=0.001, ema_momentum=0.99)

model.compile(loss=’binary_crossentropy’,optimizer=opt,metrics=[‘accuracy’])

#

# #The python script for labeling a dataframe

# Provide the interacting residue number for the human proteins

Human_loc=[69,70,73,96,97,98,101,102,104,106,107,112]

#provide the interacting residue number for the virus protein

value=[[186, 187,189],[186,189],[124,125,188,189],[121,187],[183,184,187,183,179,180],[121, 120,119,121,108,187, 184,119],[110,180],[110,109,108,119,117,118,180],[108],[106,107,108],[106,121],[187]]

#Subtract or add number in the list comprehension to get the actual residue number of virus protein

lst= [j-83 for i in value for j in i]

from itertools import islice

def convert(lst, var_lst):

idx = 0

for var_len in var_lst:

yield lst[idx : idx + var_len]

idx += var_len

#Numbers in Virus protein sublist

var_lst = [3,2,4,2,6,8,2,7,1,3,2,1]

lst1=list(convert(lst, var_lst))

print(lst1)

test1={}

for i in range(len(Human_loc)):

for j in range(len(lst1[i])):

#Subtract or add numbers to get the actual residue number of human protein

test1.update({Human_loc[i]-49:lst1[i]})

print(test1)

Li1=[]

len_vir=140

for i in test1:

#print(len(test1[i]))

for j in range(len(test1[i])):

Li1.append((i-1)*len_vir+test1[i][j])

print(Li1)

Li1.sort()

print(len(Li1))

import pandas as pd

df = pd.read_csv("2RHK_test_df.csv")

b=df.iloc[:,[0]]

print(len(b))

j=0

for i in range(len(b)):

for j in range(len(Li1)):

if Li1[j]==df.iloc[i,0]:

df.iloc[i,1]=1

break

for i in range(len(df)):

if df.loc[i,'rsa.m$value']==0 and df.loc[i,'inf.m$value']==1:

df.loc[i,'inf.m$value']=0

df.to_csv("2RHK_test_df.csv")

**Supplementary Table 2: Dataset statistics before and after SMOTE of all Protein complexes in the training set.**

| **Dataset** | **Class** | **Before Sampling** | **After Sampling** |
| --- | --- | --- | --- |
| Training | Interface (1) | 2568 | 5532150 |
| Training | Non-interface (0) | 11063699 | 5534117 |
| Ratio | 1:0 | 0.000232:1 | 0.99:1 |

**Table 7: Evaluation metrics on 16 test datasets**

| **PDB Ids** | **Virus** | **Model accuracy** | **Precision** | **Recall** | **F1-Score** |
| --- | --- | --- | --- | --- | --- |
| 7UC5 | Influenza Virus | 61% | 0.99 | 0.61 | 0.76 |
| 7T0O | HIV | 62% | 0.99 | 0.62 | 0.76 |
| 7B7N | Human Herpes virus | 63% | 0.99 | 0.63 | 0.77 |
| 6I2M | Vaccinia Virus | 61% | 0.99 | 0.60 | 0.75 |
| 5IRO | Adenovirus | 87% | 0.99 | 0.87 | 0.93 |
| 2JDQ | Influenza Virus | 92% | 0.99 | 0.92 | 0.95 |
| 3TO2 | SARS-CoV2 | 98% | 0.99 | 0.98 | 0.99 |
| 6BVV | Nipah Virus | 94% | 0.99 | 0.94 | 0.96 |
| 7KDT | SARS-CoV2 | 96% | 0.99 | 0.95 | 0.97 |
| 6E5U | Influenza virus | 97% | 0.99 | 0.96 | 0.98 |
| 6JO8 | Chikungunya | 88% | 0.99 | 0.87 | 0.93 |
| 7M4R | SARS-CoV2 | 85% | 0.99 | 0.85 | 0.92 |
| 7P9W | Epstein bar virus | 87% | 0.99 | 0.87 | 0.93 |
| 4U0A | HIV | 94% | 0.99 | 0.93 | 0.96 |
| 4U0C | HIV | 97% | 0.99 | 0.97 | 0.98 |
| 1Q94 | HIV | 87% | 0.99 | 0.86 | 0.92 |

**PDB ID 7UC5**

| **Human Protein (HLA class I histocompatibility antigen) Chain A** | **Virus Protein (Nucleoprotein peptide (Influenza Virus) Chain C** | **Distance (Å)** |
| --- | --- | --- |
| **TYR59D** | ILE61F | 4.11 |
| **ASN66D** | ARG63F | 4.12 |
| **GLN70D** | VAL66F | 4.05 |
| **THR73D** | HIS68F | 4.16 |
| **VAL76D** | HIS68F | 3.65 |
| **ASP77D** | HIS68F | 3.57 |
| **ASP77D** | LYS69F | 4.95 |
| **THR80D** | LYS69F | 4.36 |
| **TYR99D** | ARG63F | 5.9 |
| **LYS146D** | LYS69F | 4.14 |
| **GLU152D** | ARG63F | 4.18 |
| **GLU152D** | ALA67F | 4.61 |
| **LEU156D** | ARG63F | 3.91 |
| **TYR159D** | ARG63F | 3.83 |
| **TYR159D** | GLY64F | 5.15 |
| **TYR159D** | ILE61F | 3.52 |
| **TRP167D** | ILE61F | 4.37 |
| **ASP116D** | LYS69F | 3.57 |

| **Human Protein (HLA class I histocompatibility antigen)** | **Virus Protein (Nucleoprotein peptide (Influenza Virus)** | **Distance (Å)** |
| --- | --- | --- |
| **GLU63D** | ILE61F | 4.75 |
| **ASN66D** | GLY64F | 4.12 |
| **GLN70D** | VAL66F | 4.05 |
| **THR73D** | VAL66F | 4.18 |
| **THR73D** | ALA67F | 4.99 |
| **THR143D** | LYS69F | 4.19 |

**PDB ID 7T0O**

| **Human Protein (CD4)** | **Virus Protein (gp140)** | **Distance (Å)** |
| --- | --- | --- |
| **GLN25L** | LYS252D | 5.74 |
| **GLN25L** | ALA251D | 6 |
| **HIS27L** | ALA251D | 4.79 |
| **HIS27L** | ASN249D | 5.23 |
| **HIS27L** | LYS252D | 3.32 |
| **ASN32L** | SER430D | 5.45 |
| **ASN32L** | GLY429D | 5.3 |
| **ILE34L** | GLY428D | 5.42 |
| **ILE34L** | GLY429D | 3.96 |
| **ILE34L** | SER430D | 5.07 |
| **ILE34L** | THR431D | 5.28 |
| **LYS35L** | ASN250D | 3.97 |
| **LYS35L** | GLY428D | 5.73 |
| **LYS35L** | ASN249D | 5.87 |
| **LYS35L** | ALA251D | 5.52 |
| **GLN40L** | ASP444D | 2.65 |
| **GLN40L** | VAL341D | 6.28 |
| **GLN40L** | GLY443D | 5.09 |
| **GLY41L** | ASP474D | 3.9 |
| **SER42L** | ARG399D | 6.06 |
| **SER42L** | ILE400D | 4.18 |

**PDB ID 7B7N**

| **Human Protein Ephrin type A receptor 2** | **Virus Protein glycoprotein** | **Distance (Å)** |
| --- | --- | --- |
| **ARG70E** | PHE30H | 6.26 |
| **ARG70E** | GLU29H | 4.09 |
| **SER74E** | PHE30H | 6.36 |
| **PHE75E** | GLU29H | 5.54 |
| **PHE75E** | PHE30H | 5.58 |
| **PRO76E** | PHE30H | 4.9 |
| **PRO76E** | ASN31H | 4.34 |
| **PRO76E** | GLY32H | 3.8 |
| **PRO76E** | GLU29H | 4.97 |

**PDB ID 6I2M**

| **Human protein Cullin** | **Virus protein A55** | **Distance** |
| --- | --- | --- |
| **ASN27B** | ASN53A | 3.57 |
| **ASN27B** | PHE54A | 4.13 |
| **ASN27B** | ASN52A | 5.65 |
| **SER28B** | ASN53A | 4.12 |
| **SER28B** | PHE54A | 5.91 |
| **SER28B** | ILE55A | 3.78 |
| **LEU30B** | PHE54A | 4.28 |
| **LEU30B** | ILE55A | 3.73 |
| **SER31B** | ILE55A | 3.86 |
| **SER31B** | ASP56A | 4.44 |
| **SER31B** | PHE54A | 4.8 |
| **PHE32B** | PHE54A | 4.22 |
| **PHE32B** | ASP56A | 2.96 |
| **PHE32B** | LEU49A | 5.24 |
| **PHE32B** | ILE48A | 4.71 |
| **PHE32B** | TYR45A | 4.88 |
| **GLU33B** | ASP56A | 3.39 |
| **GLU33B** | TYR45A | 3.84 |
| **GLU33B** | LEU49A | 3.73 |
| **GLU33B** | VAL62A | 4.33 |
| **GLU33B** | ASN63A | 4.29 |
| **TYR36B** | TYR45A | 4.47 |
| **TYR36B** | PHE100A | 3.87 |
| **TYR36B** | ASP99A | 4.4 |
| **ARG37B** | PHE100A | 5.4 |
| **ARG37B** | HIS66A | 5.28 |
| **ARG37B** | TYR45A | 4.97 |
| **ARG37B** | ASN63A | 4.69 |
| **TYR40B** | PHE100A | 4.74 |
| **TYR40B** | HIS66A | 6.27 |
| **TYR40B** | TYR133A | 6.25 |
| **TYR40B** | ASP99A | 4.79 |
| **TYR40B** | SER96A | 3.89 |
| **LEU44B** | ASN134A | 4.42 |
| **LEU44B** | TYR133A | 5.17 |
| **LYS46B** | ASN134A | 4.23 |
| **LYS46B** | ASP131A | 3.97 |
| **LYS46B** | ALA130A | 4.64 |
| **LYS46B** | LYS132A | 4.56 |
| **MET96B** | PHE54A | 5.05 |
| **MET96B** | ASN53A | 4.44 |
| **ARG98B** | GLU44A | 4.93 |
| **ASP99B** | ILE48A | 4.53 |
| **ASP99B** | PHE54A | 5.1 |
| **ASP99B** | SER51A | 4.85 |
| **ASP99B** | ASN53A | 5.29 |
| **ILE100B** | PHE54A | 4.96 |
| **MET102B** | GLN102A | 5.48 |
| **MET102B** | ILE48A | 4.09 |
| **MET102B** | GLU44A | 5.27 |
| **MET102B** | TYR45A | 5.45 |
| **TYR103B** | GLN102A | 5.29 |
| **TYR103B** | ASP99A | 3.71 |
| **TYR103B** | PHE100A | 4.87 |
| **TYR103B** | TYR133A | 4.49 |
| **ARG106B** | GLN102A | 4.9 |
| **ARG106B** | GLU44A | 3.99 |
| **GLN111B** | LYS136A | 6.05 |
| **GLN111B** | ASN135A | 4.73 |
| **GLN111B** | LYS137A | 5.27 |
| **GLN111B** | ASN134A | 3.44 |

**PDB ID 5IRO**

| **Human Protein HLA-A2** | **Virus Protein E3** | **Distance** |
| --- | --- | --- |
| PRO50A | GLN23D | 5.1 |
| PRO50A | ALA56D | 4.62 |
| PRO50A | ALA30D | 6.19 |
| PRO50A | VAL31D | 6.19 |
| PRO50A | LEU32D | 4.91 |
| PRO50A | ASP25D | 4.36 |
| TRP51A | ALA56D | 4.01 |
| TRP51A | ASP25D | 4.08 |
| TRP51A | ALA30D | 6.4 |
| GLU53A | LEU32D | 4.83 |
| GLU53A | THR54D | 4.1 |
| GLU53A | LYS34D | 5.88 |
| GLU53A | TRP37D | 4.74 |
| GLU53A | SER21D | 5.37 |
| GLN54A | TRP37D | 4.58 |
| GLN54A | THR54D | 3.92 |
| GLN54A | ARG51D | 2.3 |
| GLN54A | LEU32D | 4.95 |
| GLN54A | LEU55D | 4.24 |
| GLN54A | ALA56D | 3.75 |
| GLN54A | LYS49D | 3.64 |
| GLU55A | TRP37D | 3.85 |
| GLU55A | ARG51D | 5.33 |
| GLY56A | TRP37D | 3.91 |
| PRO57A | TRP37D | 4.78 |
| ASN174A | LYS49D | 3.99 |
| ASN174A | ARG51D | 4.1 |
| LYS176A | LYS49D | 4.58 |
| LYS176A | ASN48D | 5.6 |
| GLU177A | ARG28D | 3.47 |
| GLU177A | THR58D | 4.14 |
| GLU177A | LYS49D | 6.26 |
| GLU177A | SER57D | 3.57 |
| GLU177A | ALA56D | 3.22 |
| THR178A | ARG28D | 3.72 |
| THR178A | ASP25D | 5.73 |
| ARG181A | ARG28D | 5.73 |
| ARG181A | PRO106D | 5.59 |
| ARG181A | GLN60D | 5.1 |
| ASP183A | MET103D | 5.78 |
| ASP183A | GLY26D | 4.97 |
| ASP183A | ASN27D | 5.76 |
| LYS186A | MET103D | 5.83 |
| LYS186A | LYS108D | 4.24 |
| SER207A | ASN27D | 5.22 |
| SER207A | MET103D | 4.55 |
| TYR209A | GLY26D | 5.44 |
| TYR209A | ASP25D | 6.28 |
| ASP238A | PRO24D | 5.63 |
| ASP238A | ASP25D | 4.19 |
| ASP238A | GLY26D | 3.09 |
| ASP238A | ASN27D | 5.71 |
| THR240A | ASN27D | 5.14 |
| THR240A | GLY26D | 4.76 |
| THR240A | MET103D | 6.16 |

**PDB ID 2JDQ**

| **Human protein Importin** | **Virus Protein PB2** | **Distance** |
| --- | --- | --- |
| SER409B | ARG692D | 5.65 |
| GLY410B | ARG692D | 4.54 |
| ALA367B | ARG692D | 5.08 |
| GLY368B | ARG692D | 5.12 |
| ASN369B | ARG692D | 6.03 |
| ARG370B | ARG692D | 5.55 |
| TRP402B | LEU695D | 4.72 |
| ASN445B | LEU695D | 3.47 |
| THR405B | LEU695D | 5.89 |
| GLN441B | LEU695D | 4.74 |
| VAL442B | LEU695D | 4.56 |
| GLN441B | LEU697D | 4.05 |
| THR506B | ARG703D | 5.48 |
| GLU509B | ARG703D | 5.81 |
| GLU507B | ARG703D | 4.21 |
| ASP508B | ARG703D | 4.56 |
| GLY284B | LYS718D | 6.13 |
| PRO285B | LYS718D | 5.21 |
| THR325B | LYS718D | 3.95 |
| ASN286B | LYS718D | 3.26 |
| LYS438B | LYS721D | 6.23 |
| GLU509B | LYS721D | 4.83 |
| TRP402B | MET735D | 4.78 |
| TRP402B | ARG737D | 4.23 |
| ALA367B | ARG737D | 4.37 |
| SER363B | ARG737D | 5.49 |
| ASN406B | ARG737D | 3.74 |
| SER409B | ARG737D | 3.97 |
| THR405B | ARG737D | 4.46 |
| ASN445B | ARG737D | 3.16 |
| ASN449B | ARG737D | 5.34 |
| ASN364B | LYS738D | 3.29 |
| ALA367B | LYS738D | 4.84 |
| THR325B | LYS738D | 4.66 |
| ASP327B | LYS738D | 5.28 |
| GLY326B | LYS738D | 3.42 |
| ASP328B | LYS738D | 4.46 |
| VAL324B | LYS738D | 2.96 |
| THR331B | LYS738D | 3.93 |
| ILE365B | LYS738D | 5.11 |
| GLN372B | LYS738D | 5.55 |
| THR325B | ARG739D | 4.64 |
| ASN364B | ARG739D | 2.91 |
| TRP360B | ARG739D | 3.78 |
| TRP402B | ARG739D | 5.05 |
| SER363B | ARG739D | 4.35 |
| GLU399B | ARG739D | 2.99 |
| THR325B | ASP740D | 4.95 |
| ASN322B | ASP740D | 4.11 |
| TRP360B | ASP740D | 3.89 |
| LYS243B | ASP740D | 4.26 |
| ASP283B | ASP740D | 5.16 |
| GLY284B | ASP740D | 3.47 |
| ASP283B | SER741D | 4.46 |
| ASN322B | SER741D | 3.71 |
| TRP360B | SER741D | 4.28 |
| ARG318B | SER741D | 5.56 |
| LYS243B | SER741D | 3.06 |
| SER279B | SER741D | 5.7 |
| TRP276B | GLN748D | 5.28 |
| ARG241B | GLN748D | 5.2 |
| ARG318B | GLN748D | 3.36 |
| TYR280B | GLN748D | 5.06 |
| ARG241B | THR749D | 4.36 |
| TRP276B | THR749D | 5.28 |
| TYR280B | THR749D | 3.86 |
| ARG241B | ALA750D | 4.17 |
| TRP234B | ALA750D | 4.8 |
| ASN238B | ALA750D | 5.38 |
| ASN238B | THR751D | 4.6 |

**PDB ID 3TO2**

| **Human protein HLA-A*0201** | **Virus protein M** | **Distance** |
| --- | --- | --- |
| TYR7A | ALA2C | 4.63 |
| TYR7A | LEU1C | 4.15 |
| TYR59A | LEU1C | 5.92 |
| GLU63A | LEU1C | 4.35 |
| GLU63A | ALA2C | 2.79 |
| ARG65A | PHE4C | 4.74 |
| LYS66A | PHE4C | 4.97 |
| LYS66A | ALA2C | 5.09 |
| LYS66A | LEU6C | 5.34 |
| LYS66A | CYS3C | 4.26 |
| ALA69A | LEU6C | 4.45 |
| ALA69A | PHE4C | 4.2 |
| HIS70A | LEU6C | 4.03 |
| HIS70A | ALA2C | 4.72 |
| HIS70A | CYS3C | 2.95 |
| HIS70A | VAL5C | 5.44 |
| THR73A | ALA8C | 5.92 |
| THR73A | LEU6C | 4.55 |
| THR73A | ALA7C | 4.32 |
| ASP77A | VAL9C | 4.1 |
| ASP77A | ALA8C | 3.49 |
| THR80A | VAL9C | 4.46 |
| LEU81A | VAL9C | 3.8 |
| TYR84A | VAL9C | 4.81 |
| ARG97A | LEU6C | 5.21 |
| ARG97A | VAL5C | 4.83 |
| ARG97A | ALA7C | 4.73 |
| TYR99A | CYS3C | 4.96 |
| TYR99A | ALA2C | 4.61 |
| TYR99A | VAL5C | 5.91 |
| TYR99A | LEU6C | 6.1 |
| HIS114A | CYS3C | 5.67 |
| HIS114A | VAL5C | 5.02 |
| TYR116A | VAL9C | 4.91 |
| THR143A | VAL9C | 4.38 |
| LYS146A | ALA8C | 6.3 |
| LYS146A | VAL9C | 3.91 |
| TRP147A | ALA7C | 4.39 |
| TRP147A | ALA8C | 3.72 |
| TRP147A | VAL9C | 5.38 |
| VAL152A | VAL5C | 5.02 |
| VAL152A | ALA7C | 4.25 |
| GLN155A | VAL5C | 3.96 |
| GLN155A | CYS3C | 5.6 |
| LEU156A | CYS3C | 5.36 |
| LEU156A | VAL5C | 4.69 |
| TYR159A | CYS3C | 4.09 |
| TYR159A | VAL5C | 5.4 |
| TYR159A | ALA2C | 4.05 |

**PDB ID 6BVV**

| **Human Importin** | **Virus W protein** | **Distance (Å)** |
| --- | --- | --- |
| SER100A | ARG440B | 3.87 |
| SER100A | VAL441B | 5.15 |
| SER100A | VAL441B | 4.28 |
| SER100A | VAL441B | 5.35 |
| SER100A | SER442B | 4.35 |
| SER100A | VAL441B | 5.37 |
| SER100A | SER442B | 3.45 |
| SER100A | SER442B | 4.14 |
| SER100A | ARG440B | 4.78 |
| SER100A | VAL441B | 4.59 |
| SER100A | SER442B | 4.53 |
| SER100A | SER442B | 3.51 |
| SER100A | SER442B | 4.09 |
| SER101A | SER442B | 5.23 |
| SER101A | SER442B | 5.6 |
| SER101A | SER442B | 3.74 |
| SER101A | VAL441B | 4.46 |
| SER101A | SER442B | 4.47 |
| SER101A | SER442B | 5.48 |
| ARG103A | VAL441B | 4.28 |
| ARG103A | VAL441B | 4.31 |
| ARG103A | VAL441B | 2.78 |
| ARG103A | VAL441B | 3.69 |
| ARG103A | VAL441B | 5.04 |
| PHE133A | ARG440B | 5.48 |
| PHE133A | ARG440B | 3.81 |
| PHE133A | ARG440B | 4.69 |
| PHE133A | ARG440B | 3.69 |
| PHE133A | ARG440B | 4.62 |
| PHE133A | ARG440B | 4.07 |
| TRP137A | ARG440B | 5.11 |
| TRP137A | ARG440B | 3.85 |
| TRP137A | ARG440B | 4.6 |
| TRP137A | ARG440B | 4.51 |
| TRP137A | ARG440B | 4.24 |
| TRP137A | ARG440B | 2.88 |
| TRP137A | ARG440B | 3.81 |
| TRP137A | ARG440B | 4.97 |
| TRP137A | ARG440B | 6.07 |
| TRP137A | ARG440B | 5.84 |
| TRP137A | ARG440B | 3.99 |
| TRP137A | ARG440B | 4.5 |
| TRP137A | ARG440B | 4.06 |
| TRP137A | ARG440B | 4.81 |
| TRP137A | ARG440B | 4.93 |
| TRP137A | ARG440B | 4.07 |
| TRP137A | ARG440B | 4.79 |
| TRP137A | ARG440B | 4.5 |
| TRP137A | ARG440B | 5.14 |
| TRP137A | VAL441B | 4.11 |
| TRP137A | VAL441B | 3.68 |
| TRP137A | SER442B | 5.51 |
| TRP137A | SER442B | 5.5 |
| TRP137A | SER442B | 4.55 |
| TRP137A | ARG440B | 4.88 |
| TRP137A | ARG440B | 4.98 |
| TRP137A | ARG440B | 3.38 |
| TRP137A | ARG440B | 3.77 |
| TRP137A | ARG440B | 4.74 |
| TRP137A | ARG440B | 5.25 |
| TRP137A | SER442B | 4.91 |
| TRP137A | SER442B | 5.73 |
| ASN141A | ALA439B | 4.71 |
| ASN141A | ALA439B | 5.27 |
| ASN141A | ALA439B | 5.31 |
| ASN141A | ARG440B | 4.36 |
| ASN141A | ALA439B | 4.89 |
| ASN141A | ARG440B | 5.12 |
| ASN141A | ARG440B | 5.2 |
| ASN141A | ARG440B | 3.79 |
| ASN141A | ARG440B | 4.21 |
| SER144A | LYS437B | 5.54 |
| SER144A | LYS437B | 4.11 |
| SER144A | LYS437B | 4.33 |
| SER144A | LYS437B | 2.96 |
| SER144A | LYS438B | 4.89 |
| SER144A | LYS438B | 5.68 |
| SER144A | ALA439B | 4.63 |
| SER144A | LYS437B | 4.34 |
| SER144A | LYS438B | 3.58 |
| SER144A | LYS438B | 4.63 |
| SER144A | LYS438B | 4.23 |
| SER144A | LYS438B | 4.3 |
| SER144A | ALA439B | 4.18 |
| SER144A | ALA439B | 4.99 |
| SER144A | ALA439B | 4.18 |
| SER144A | ALA439B | 4.1 |
| SER144A | ALA439B | 6.31 |
| TRP179A | LYS438B | 4.21 |
| TRP179A | ARG440B | 5.96 |
| TRP179A | LYS438B | 4.57 |
| TRP179A | LYS438B | 5.2 |

**PDB ID 7KDT**

| **Human TOM70** | **Virus ORF9b** | **Distance** |
| --- | --- | --- |
| ILE215A | ALA75B | 5.55 |
| ILE215A | ILE74B | 5.31 |
| PHE219A | GLN70B | 4.95 |
| PHE219A | PHE69B | 4.52 |
| PHE219A | ALA75B | 5.47 |
| MET225A | LYS40B | 6.21 |
| MET225A | GLN70B | 5.19 |
| ASP229A | LYS40B | 5.3 |
| PHE256A | SER50B | 5.75 |
| PHE256A | PRO51B | 6.34 |
| PHE256A | LEU54B | 5.77 |
| PHE256A | ARG47B | 5.31 |
| PHE256A | LEU48B | 3.99 |
| PHE256A | GLY49B | 5.71 |
| SER259A | GLY49B | 4.41 |
| SER259A | SER50B | 5.49 |
| SER259A | PRO51B | 4.94 |
| TYR260A | GLY49B | 4.19 |
| TYR260A | LEU48B | 3.81 |
| SER263A | GLY49B | 4.85 |
| SER263A | SER50B | 5.57 |
| LEU340A | LEU48B | 3.38 |
| LEU341A | LEU48B | 4.09 |
| SER375A | LEU46B | 4.78 |
| SER375A | LEU48B | 5.52 |
| MET378A | LEU46B | 5.39 |
| MET378A | ILE44B | 5.02 |
| GLN379A | LEU48B | 6.34 |
| GLN379A | LEU46B | 4.32 |
| GLN379A | ILE45B | 4.9 |
| GLN379A | ARG47B | 4.82 |
| GLN381A | PRO43B | 5.47 |
| GLN381A | ILE44B | 3.77 |
| GLN409A | LEU52B | 4.71 |
| GLN409A | SER50B | 4.69 |
| GLN409A | GLY49B | 5.77 |
| GLN409A | PRO51B | 5.01 |
| GLN409A | ASN55B | 5.84 |
| LEU410A | LEU46B | 5.83 |
| LEU410A | ILE44B | 5.12 |
| ILE412A | MET56B | 5.37 |
| ILE412A | LYS59B | 5.43 |
| ILE412A | LEU52B | 3.77 |
| LEU413A | MET56B | 5.29 |
| LEU413A | LYS59B | 5.22 |
| LEU413A | ILE44B | 4.65 |
| LEU413A | LEU46B | 4.54 |
| LEU413A | LEU52B | 4.3 |
| LEU413A | ASN55B | 3.79 |
| LEU414A | ILE44B | 4.7 |
| ASP415A | LYS59B | 5.73 |
| GLN440A | LEU52B | 5.28 |
| PHE443A | LEU52B | 4.95 |
| PHE443A | MET56B | 3.74 |
| ARG447A | MET56B | 3.89 |
| ARG447A | LYS59B | 5.28 |
| GLU477A | PRO51B | 5.37 |
| GLU477A | SER53B | 3.78 |
| GLU477A | LEU52B | 3.23 |
| LEU481A | SER53B | 4.25 |
| LEU481A | LEU52B | 5.14 |
| LEU481A | MET56B | 5.57 |
| GLN484A | ALA57B | 6.06 |
| GLN484A | SER53B | 3.97 |
| GLN484A | MET56B | 5.49 |
| HIS515A | SER53B | 5.57 |
| HIS515A | LEU54B | 4.81 |
| HIS515A | ALA57B | 3.61 |
| LEU518A | LEU64B | 5.09 |
| LEU518A | THR60B | 6.37 |
| LEU518A | ALA57B | 5.62 |
| LEU518A | LEU61B | 5.23 |
| LEU521A | LEU64B | 5.05 |
| LEU521A | LEU61B | 5.94 |
| GLN522A | LEU64B | 5.73 |
| GLN522A | THR60B | 4.83 |
| ASP545A | ARG47B | 3.68 |
| ASP545A | ARG58B | 4.33 |
| PHE546A | ARG47B | 4.36 |
| PHE546A | LEU54B | 4.56 |
| PHE546A | ARG58B | 4.73 |
| PHE546A | LEU61B | 3.79 |
| PHE546A | ALA57B | 3.94 |
| GLU549A | LEU61B | 4.34 |
| GLU549A | GLU65B | 3.32 |
| GLU549A | ARG47B | 5.08 |
| GLU549A | ARG58B | 4.42 |
| THR553A | ALA68B | 4.5 |
| THR553A | LEU64B | 5.03 |
| THR553A | GLU65B | 4.79 |
| THR553A | LEU61B | 4.2 |
| VAL556A | ALA68B | 4.36 |
| VAL556A | THR72B | 3.37 |
| VAL556A | PHE69B | 4.06 |
| GLN557A | ALA68B | 4.35 |
| GLN557A | LEU71B | 3.51 |
| GLN557A | THR72B | 4.43 |
| GLN557A | PRO73B | 3.66 |
| GLN557A | LEU64B | 5.3 |
| GLN557A | LYS67B | 5.74 |
| ARG558A | PRO73B | 5.21 |
| GLY559A | THR72B | 3.65 |
| GLY559A | PRO73B | 5.26 |

**PDB ID 6E5U**

| **Human NXF1** | **Virus NS1** | **Distance** |
| --- | --- | --- |
| LYS213A | ILE137U | 5.71 |
| LYS213A | PHE138U | 4.41 |
| LEU214A | ILE137U | 6.07 |
| MET216A | PHE138U | 4.01 |
| SER217A | PHE138U | 3.58 |
| SER217A | ILE137U | 5.15 |
| SER217A | VAL136U | 5.33 |
| SER217A | ASP139U | 5.48 |
| TYR220A | PHE138U | 4.06 |
| TYR220A | ASP139U | 5.71 |
| ASN263A | PHE138U | 5.56 |
| ASN263A | ARG140U | 5.63 |

**PDB ID 6JO8**

| **Human protein MXRA8** | **Virus Envelope** | **Distance** |
| --- | --- | --- |
|  |  |  |
| LEU237M | GLU39B | 5.81 |
| LEU237M | THR37B | 5.99 |
| LEU237M | SER130B | 6.16 |
| LEU237M | ARG267B | 6.35 |
| LEU237M | PRO265B | 4.57 |
| PHE238M | THR145B | 4.9 |
| PHE238M | SER130B | 5.22 |
| PHE238M | ALA146B | 6.35 |
| PHE238M | TYR147B | 5.38 |
| ARG240M | PRO265B | 4.13 |
| ARG240M | ARG267B | 5.42 |
| ARG240M | THR263B | 5.27 |
| ARG240M | ASN264B | 2.88 |
| ASP241M | LYS132B | 5.49 |
| ASP241M | THR145B | 5.13 |

**PDB ID 7M4R**

| **Human protein PALS1** | **Virus envelope protein** | **Distance** |
| --- | --- | --- |
| PRO266A | VAL75C | 5.38 |
| PRO266A | LEU74C | 3.61 |
| LEU267A | VAL75C | 4.79 |
| GLY268A | VAL75C | 5.31 |
| GLY268A | LEU74C | 5.2 |
| ALA269A | VAL75C | 3.99 |
| ALA269A | LEU74C | 3.73 |
| THR270A | LEU73C | 3.53 |
| THR270A | ASP72C | 5.77 |
| THR270A | LEU74C | 5.18 |
| VAL271A | LEU73C | 3.1 |
| VAL271A | ASP72C | 4.33 |
| VAL271A | VAL75C | 4.03 |
| ARG272A | ASP72C | 4.15 |
| ARG272A | ARG69C | 6.32 |
| ARG272A | PRO71C | 5.12 |
| SER281A | ASP72C | 5.25 |
| VAL314A | LEU73C | 4.3 |
| VAL314A | ASP72C | 6.21 |
| PHE318A | LEU73C | 4.72 |
| PHE318A | VAL75C | 5.24 |
| PHE318A | LEU74C | 3.57 |
| LEU321A | VAL75C | 5.01 |
| PRO365A | PRO71C | 5.75 |
| PRO365A | ASP72C | 4.56 |
| CYS366A | PRO71C | 4.86 |
| CYS366A | ASP72C | 5.73 |
| CYS366A | LEU73C | 5.14 |
| CYS366A | LEU74C | 4.82 |
| GLU368A | LEU74C | 5.41 |
| GLU368A | LEU73C | 5.99 |
| LEU369A | LEU74C | 4.24 |

**PDB ID 7P9W**

| **Human BCL2 binding component** | **Virus protein BHRF1** | **Distance** |
| --- | --- | --- |
| GLN132B | ARG71A | 4.52 |
| TRP133B | ARG71A | 4.27 |
| TRP133B | PHE72A | 5.17 |
| TRP133B | HIS75A | 4.66 |
| TRP133B | THR76A | 4.87 |
| TRP133B | VAL86A | 5.09 |
| TRP133B | HIS78A | 5.76 |
| TRP133B | ASP82A | 3.71 |
| TRP133B | SER85A | 6.09 |
| ALA134B | VAL86A | 4.11 |
| ALA134B | GLU89A | 5.18 |
| ALA134B | SER85A | 5.19 |
| ARG135B | GLU89A | 5.31 |
| ARG135B | HIS92A | 4.93 |
| GLU136B | ARG71A | 5.23 |
| GLU136B | GLU67A | 5.95 |
| GLU136B | THR68A | 5.53 |
| ILE137B | THR68A | 4.39 |
| ILE137B | VAL86A | 4.95 |
| ILE137B | ARG71A | 5.54 |
| ILE137B | TRP107A | 4.73 |
| ILE137B | PHE72A | 4.1 |
| GLY138B | VAL86A | 4.81 |
| GLY138B | GLU89A | 4.97 |
| GLY138B | ILE90A | 3.65 |
| GLN140B | THR64A | 4.11 |
| GLN140B | THR68A | 4.4 |
| LEU141B | ALA103A | 4.68 |
| LEU141B | ILE90A | 5.09 |
| LEU141B | ARG100A | 4.98 |
| LEU141B | TRP104A | 5.48 |
| LEU141B | THR68A | 6.03 |
| LEU141B | TRP107A | 4.52 |
| LEU141B | VAL86A | 4.71 |
| LEU141B | PHE65A | 4.48 |
| ARG142B | ILE90A | 4.97 |
| ARG142B | ARG100A | 4.5 |
| ARG142B | GLU89A | 3.92 |
| ARG142B | EDO210A | 4.81 |
| ARG142B | HIS92A | 4.07 |
| MET144B | ASN61A | 5.13 |
| MET144B | CL220A | 5.47 |
| MET144B | ALA103A | 4.83 |
| MET144B | THR64A | 5.22 |
| MET144B | PHE65A | 4.1 |
| MET144B | ILE57A | 3.88 |
| ALA145B | GLY99A | 3.67 |
| ALA145B | ALA103A | 4.21 |
| ALA145B | ARG100A | 3.13 |
| ASP146B | SER97A | 5.72 |
| ASP146B | ARG100A | 3.79 |
| LEU148B | CL220A | 4.77 |
| LEU148B | ILE57A | 5.35 |
| LEU148B | GLY99A | 4.5 |
| LEU148B | LEU53A | 6.2 |
| LEU148B | LEU102A | 4.87 |
| LEU148B | LEU98A | 5.06 |
| ASN149B | SER97A | 5.29 |
| ASN149B | GLY99A | 3.87 |
| ASN149B | LEU98A | 4 |
| ASN149B | NH4208A | 5.37 |

**PDB ID 4U0C**

| **Human Protein Nup153** | **Virus Protein Capsid** | **Distance** |
| --- | --- | --- |
| **ASN53A** | PHE1417B | 4.34 |
| **ASN53A** | GLY1418B | 4.41 |
| **LEU56A** | PHE1417B | 4.13 |
| **ASN57A** | PHE1417B | 4.22 |
| **ASN57A** | THR1416B | 6.06 |
| **ASN57A** | PHE1415B | 5.02 |
| **ASN57A** | VAL1414B | 4.87 |
| **MET66A** | PHE1417B | 4.19 |
| **MET66A** | THR1416B | 5.54 |
| **LEU69A** | PHE1417B | 4.42 |
| **LYS70A** | PHE1417B | 3.94 |
| **LYS70A** | THR1416B | 4.99 |
| **THR107A** | GLY1418B | 3.68 |
| **ILE73A** | PHE1417B | 5.6 |
| **GLN67A** | THR1416B | 4.1 |
| **GLN63A** | THR1416B | 4.6 |
| **THR54A** | PHE1415B | 3.7 |
| **GLY106A** | GLY1418B | 5.6 |
| **TYR130** | PHE1417B | 6.4 |

**PDB ID 4U0A**

| **Human peptide CPSF6** | **Virus protein capsid** | **Distance** |
| --- | --- | --- |
| PRO313B | ASN74A | 5.99 |
| VAL314B | ASN74A | 5.7 |
| VAL314B | SER102A | 5.4 |
| VAL314B | THR107A | 5.27 |
| VAL314B | ILE73A | 5.2 |
| VAL314B | ALA77A | 4.52 |
| VAL314B | ALA105A | 3.85 |
| VAL314B | GLY101A | 5.08 |
| LEU315B | ASN74A | 2.79 |
| LEU315B | LYS70A | 5.14 |
| LEU315B | GLN67A | 5.87 |
| LEU315B | ILE73A | 4.87 |
| LEU315B | ALA105A | 5.67 |
| LEU315B | THR107A | 3.92 |
| PHE316B | GLN67A | 4.6 |
| PHE316B | LYS70A | 4.41 |
| PRO317B | GLN67A | 4.08 |
| PRO320B | ASN57A | 3.62 |
| PRO320B | LYS70A | 5.15 |
| PHE321B | ASN57A | 2.97 |
| PHE321B | TYR130A | 5.13 |
| PHE321B | ASN53A | 3.92 |
| PHE321B | LEU56A | 4.41 |
| PHE321B | LYS70A | 5.19 |
| PHE321B | ILE73A | 4.23 |
| PHE321B | MET66A | 4.24 |
| PHE321B | LEU69A | 4.81 |
| PHE321B | GLN63A | 6.09 |
| GLY322B | ASN53A | 3.57 |
| GLY322B | ALA105A | 4.39 |

**PDB ID: 1Q94**

| **Human protein HLA-A*1101** | **Virus Protein reverse transcriptase** | **Distance** |
| --- | --- | --- |
| TYR7A | ILE2C | 3.73 |
| TYR7A | ALA1C | 4.12 |
| GLU63A | ILE2C | 4.8 |
| GLU63A | ALA1C | 2.9 |
| ASN66A | ILE2C | 3.66 |
| ASN66A | GLN4C | 5.24 |
| ASN66A | PHE3C | 5.37 |
| VAL67A | ILE2C | 3.73 |
| GLN70A | SER6C | 5.59 |
| GLN70A | ILE2C | 5.5 |
| GLN70A | PHE3C | 5.19 |
| THR73A | THR8C | 5 |
| THR73A | SER6C | 4.46 |
| THR73A | MET7C | 4.16 |
| VAL76A | THR8C | 5.15 |
| ASP77A | LYS9C | 5.01 |
| ASP77A | THR8C | 4.37 |
| TYR159A | ALA1C | 5.21 |
| ARG163A | ALA1C | 5.49 |
| ARG163A | ILE2C | 5.77 |
| TRP167A | ALA1C | 2.94 |
| TYR171A | ALA1C | 4.76 |
