# Supplementary material for: HVIface: sequence-based deep learning for decoding human-virus protein-protein interfaces
Source: Front Bioinform. 2026 May 8;6:1813796. doi: 10.3389/fbinf.2026.1813796 (PMC13194012; doi:10.3389/fbinf.2026.1813796)
Supplement: Supplementary file 2 [file Table2.docx]

**Supplementary Table 1. List of 73 Human-Virus proteins**

| PDB IDs | Human Protein | Virus Protein | Virus Name |
| --- | --- | --- | --- |
| 5FW5^1^ | RAS GTPASE-ACTIVATING PROTEIN-BINDING PROTEIN 1 | NON-STRUCTURAL PROTEIN 3 | Semliki Forest Virus |
| 5I22^1^ | Myc box-dependent-interacting protein 1 | CHIKV nsP3 peptide | Chikungunya Virus |
| 6JO8^2^ | Matrix remodelling-associated protein 8 | E1 | Chikungunya Virus |
| 5WJN^1^ | HLA class I histocompatibility antigen, A-11 alpha chain | GTS3 peptide | Dengue Virus |
| 7T0O^2^ | T-cell surface glycoprotein CD4 | BG505 SOSIP.664 gp140 | HIV |
| 4LQW^1^ | E3 SUMO-protein ligase RanBP2 | Capsid protein p24 | HIV |
| 2MA9^1^ | Transcription elongation factor B polypeptide 2 | Virion infectivity factor | HIV |
| 7R7V^1^ | MHC class I antigen | Gag-derived epitope QW9 | HIV |
| 4I48^1^ | HLA-A68 | Env-derived peptide | HIV |
| 2BVP^1^ | HLA CLASS I HISTOCOMPATIBILITY ANTIGEN, B-57 ALPHA CHAIN | P24 | HIV |
| 1A1M^1^ | HLA class I histocompatibility antigen, BW-53 B*5301 alpha chain | GAG PROTEIN | HIV |
| 4U5W^1^ | Src Family Kinase Hck SH3-SH2 | Nef-SF2 | HIV |
| 5L1Z^1^ | Cyclin-dependent kinase 9 | Protein Tat | HIV |
| 6XQI^1^ | hHR23A | Vpr | HIV |
| 7D7S^1^ | Fyn SH3 R96I mutant | SF2 Nef | HIV |
| 3REA^1^ | Hck-SH3 domain | Nef | HIV |
| 3W39^1^ | HLA-B*5201 | Gag-Pol polyprotein | HIV |
| 7UC5^2^ | HLA class I histocompatibility antigen, A alpha chain | Nucleoprotein peptide | Influenza virus |
| 2BST^1^ | HLA CLASS I HISTOCOMPATIBILITY ANTIGEN, B-27 ALPHA CHAIN PRECURSOR | NUCLEOPROTEIN | Influenza virus |
| 2RHK^1^ | Cleavage and polyadenylation specificity factor subunit 4 | Non-structural protein 1 | Influenza virus |
| 7JYW^1^ | MHC class I antigen | PB2 | Influenza virus |
| 6J2A^1^ | HLA-A*3003 | NP44 | Influenza virus |
| 5UL6^1^ | Adapter molecule crk | Proline-rich motif of nonstructural protein 1 of influenza A virus | Influenza virus |
| 6E5U^2^ | Nuclear RNA export factor 1 | Non-structural protein 1 | Influenza virus |
| 5TEZ^1^ | M1-HLA-A2 | TCR F50 | Influenza virus |
| 2X4S^1^ | HLA-A2.1 | NUCLEOPROTEIN | Influenza virus |
| 7WT4^1^ | MHC class I antigen | PB1 peptide | Influenza virus |
| 2JDQ^2^ | Importin alpha 5 | PB2 | Influenza virus |
| 7TUQ^1^ | BRD4 bromodomain 1 | Envelope small membrane protein | SARS-CoV-2 |
| 7EKF^1^ | ACE2 | spike receptor-binding domain | SARS-CoV-2 |
| 7WBL^1^ | ACE2 | Spike protein S1 | SARS-CoV-2 |
| 7MKB^1^ | Human leukocyte antigen A*0201 | Spike protein S1 | SARS-CoV-2 |
| 7OPL^1^ | DNA Polymerase alpha - primase | nsp1 | SARS-CoV-2 |
| 7SN0^1^ | ACE2 | Surface glycoprotein | SARS-CoV-2 |
| 7WBQ^1^ | ACE2 | Spike glycoprotein | SARS-CoV-2 |
| 7QIK^1^ | 14-3-3 protein sigma | Nucleocapsid phosphopeptide | SARS-CoV-2 |
| 6YXJ^1^ | Polyadenylate-binding protein-interacting protein 1 | NSP3 | SARS-CoV-2 |
| 7RBR^1^ | ubiquitin | Papain-Like Protease | SARS-CoV-2 |
| 7VX9^1^ | ACE2 | Spike glycoprotein | SARS-CoV-2 |
| 7SUO^1^ | G3BP1 NTF2-like domain | IDR1 | SARS-CoV-2 |
| 9DW6^1^ | human tRNA methyltransferase TRMT1 | nsp5 | SARS-CoV-2 |
| 5TL6^1^ | ISG15 | papain-like protease | SARS-CoV-2 |
| 7M4R^2^ | [PALS1](https://www.rcsb.org/structure/7M4R) | ENVELOPE PROTEIN | SARS-CoV-2 |
| 7T2U^1^ | NEMO | 3C-Like Protease | SARS-CoV-2 |
| 7K5I^1^ | 40S ribosomal protein S17 | nsp1 | SARS-CoV-2 |
| 7KDT^2^ | TOM70 | ORF9B | SARS-CoV-2 |
| 8CMI^1^ | Human leukocyte antigen DR beta chain allotype DR1 (DRB1*0101) | Spike protein S2' | SARS-CoV-2 |
| 7XCH^1^ | Processed angiotensin-converting enzyme 2 | Spike glycoprotein | SARS-CoV-2 |
| 3TO2^2^ | MHC class I antigen | Md3-C9 peptide derived from the Membrane glycoprotein | SARS-CoV-2 |
| 7F90^1^ | nuclear protein | auxiliary protein | SARS-CoV-2 |
| 7JJC^1^ | Spike protein S1 | Neuropilin-1 | SARS-CoV-2 |
| 1IM3^1^ | HLA-A2 | US2 | Cytomegalovirus |
| 2AXG^1^ | HLA-B*3508 | BZLF1 | Epstein-Barr virus (EBV |
| 2VSM^1^ | HEMAGGLUTININ-NEURAMINIDASE | EFNB2 | Henipavirus nipahens |
| 2X4R^1^ | HLA CLASS I HISTOCOMPATIBILITY ANTIGEN, A-2.1 | PHOSPHOPROTEIN | Human betaherpesvirus 5 |
| 2XPX^1^ | BCL-2 HOMOLOGOUS ANTAGONIST/KILLER | Bhrf1 | [human gammaherpesvirus 4](https://www.rcsb.org/search?q=rcsb_entity_source_organism.taxonomy_lineage.name:human%20gammaherpesvirus%204) |
| 2XXN^1^ | UBIQUITIN CARBOXYL-TERMINAL HYDROLASE 7 | K10 | Human gammaherpesvirus 8 |
| 3MRM^1^ | HLA class I histocompatibility antigen, A-2 alpha chain | NS3 | HCV |
| 4FA8^1^ | Macrophage colony-stimulating factor 1 | Secreted protein BARF1 | human gammaherpesvirus 4 |
| 4ZNY^1^ | Tumor susceptibility gene 101 protein | T-cell leukemia virus type I, partial gag gene; HTLV1 (human T-lymphotropic virus type I) | Human T-cell Leukemia type I virus |
| 5HDA^1^ | Zinc finger MYND domain-containing protein 11 | Epstein-Barr nuclear antigen 2 | Human herpesvirus 4 strain B95-8 |
| 5IRO^2^ | HLA class I histocompatibility antigen, A-2 alpha chain | TAX protein | Human T-cell leukemia virus type I |
| 6BVV^2^ | Importin subunit alpha-3 | Protein W | Henipavirus nipahense |
| 6I2M^2^ | Cullin-3 protein A55 | Kelch repeat and BTB domain-containing | Vaccinia virus WR |
| 7B7N^2^ | Ephrin type-A receptor 2 | Envelope glycoprotein H | Human gammaherpesvirus 8 |
| 7P9W^2^ | Bcl-2-binding component 3, isoforms ½ | Apoptosis regulator BHRF1 | [Human herpesvirus 4 strain B95-8](https://www.rcsb.org/search?q=rcsb_entity_source_organism.taxonomy_lineage.name:Human%20herpesvirus%204%20strain%20B95-8) |
| 7QS8^1^ | Protein scribble homolog | Protein Tax-1 | Human T-cell leukemia virus type I |
| 7QTX^1^ | Bcl-2-binding component 3, isoforms ½ | Bcl-2 | [Human gammaherpesvirus 8](https://www.rcsb.org/search?q=rcsb_entity_source_organism.taxonomy_lineage.name:Human%20gammaherpesvirus%208) |
| 7TI5^1^ | Dyslexia-associated protein KIAA0319-like protein | Capsid protein | [Adeno-associated virus](https://www.rcsb.org/search?q=rcsb_entity_source_organism.taxonomy_lineage.name:Adeno-associated%20virus) |
| 7WLP^1^ | Histone H2B type 1-O,Histone H2A type 1-D | Tegument protein BKRF4 | [human gammaherpesvirus 4](https://www.rcsb.org/search?q=rcsb_entity_source_organism.taxonomy_lineage.name:human%20gammaherpesvirus%204) |
| 4U0A^2^ | CPSF6 Peptide | Capsid protein | HIV |
| 4U0C^2^ | Nup153 Peptide | P24 | HIV |
| 1Q94^2^ | HLA Class I | nonamer reverse transcriptase | HIV |
|  |  |  |  |
|  |  |  |  |
|  |  |  |  |

**Superscripts:** ¹ Training dataset; ² Test dataset.
