# Supplementary material for: HVIface: sequence-based deep learning for decoding human-virus protein-protein interfaces
Source: Front Bioinform. 2026 May 8;6:1813796. doi: 10.3389/fbinf.2026.1813796 (PMC13194012; doi:10.3389/fbinf.2026.1813796)
Supplement: Supplementary file 3 [file Table3.docx]

**Supplementary Table 2: Dataset statistics before and after SMOTE of all Protein complexes in the training set.**

| **Dataset** | **Class** | **Before Sampling** | **After Sampling** |
| --- | --- | --- | --- |
| Training | Interface (1) | 2568 | 5532150 |
| Training | Non-interface (0) | 11063699 | 5534117 |
| Ratio | 1:0 | 0.000232:1 | 0.99:1 |
