# Supplementary material for: HVIface: sequence-based deep learning for decoding human-virus protein-protein interfaces
Source: Front Bioinform. 2026 May 8;6:1813796. doi: 10.3389/fbinf.2026.1813796 (PMC13194012; doi:10.3389/fbinf.2026.1813796)
Supplement: Supplementary file 4 [file Supplementaryfile1.pptx]

## Slide 1
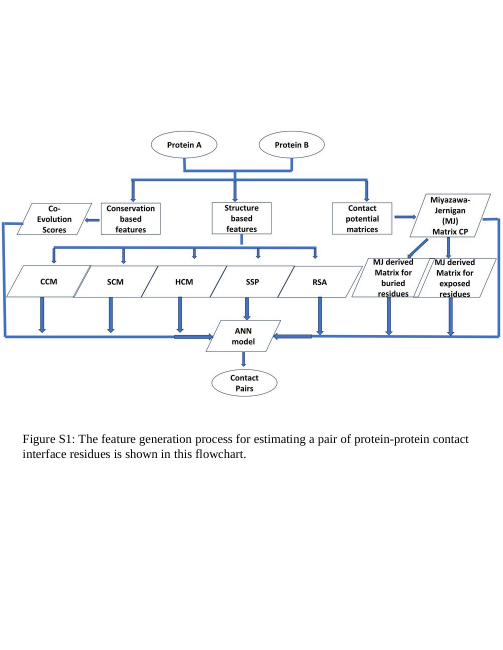

Figure S1: The feature generation process for estimating a pair of protein-protein contact interface residues is shown in this flowchart.

## Slide 2
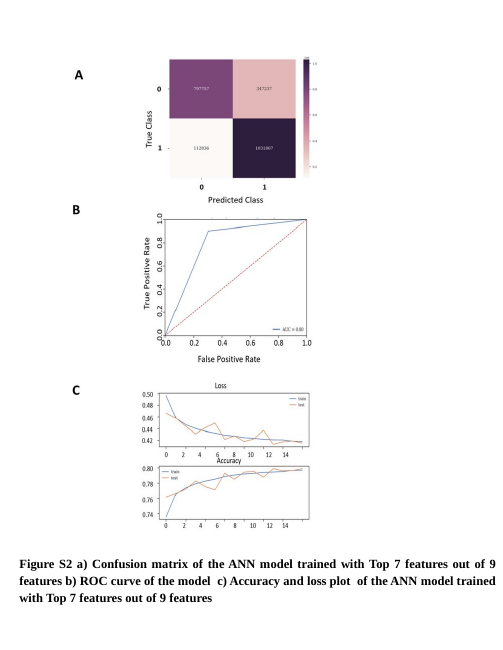

Figure S2 a) Confusion matrix of the ANN model trained with Top 7 features out of 9 features b) ROC curve of the model c) Accuracy and loss plot of the ANN model trained with Top 7 features out of 9 features

## Slide 3
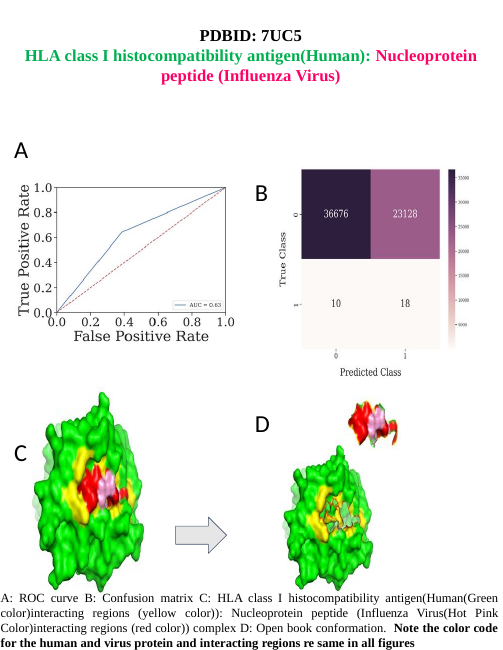

PDBID: 7UC5
HLA class I histocompatibility antigen(Human): Nucleoprotein peptide (Influenza Virus)
A
B
D
C
A: ROC curve B: Confusion matrix C: HLA class I histocompatibility antigen(Human(Green color)interacting regions (yellow color)): Nucleoprotein peptide (Influenza Virus(Hot Pink Color)interacting regions (red color)) complex D: Open book conformation. Note the color code for the human and virus protein and interacting regions re same in all figures

## Slide 4
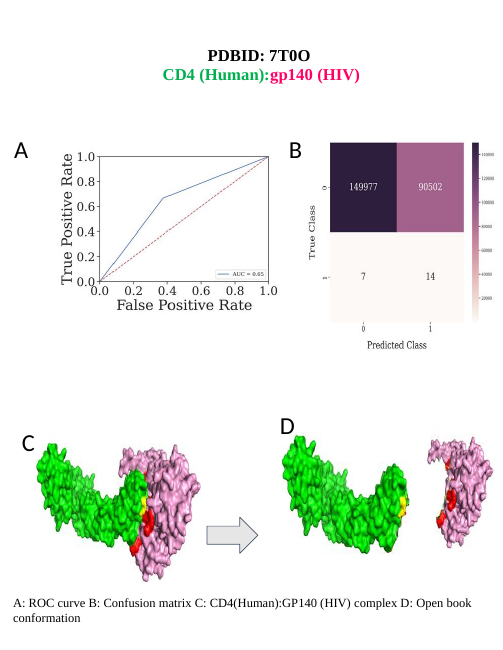

PDBID: 7T0O
 CD4 (Human):gp140 (HIV)
A
B
D
C
A: ROC curve B: Confusion matrix C: CD4(Human):GP140 (HIV) complex D: Open book conformation

## Slide 5
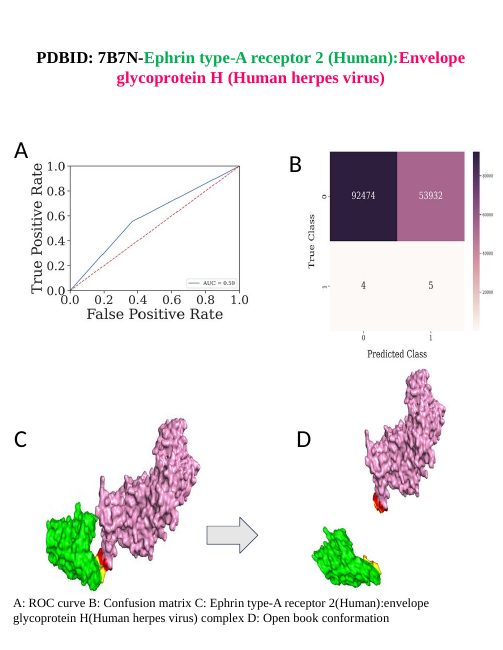

PDBID: 7B7N-Ephrin type-A receptor 2 (Human):Envelope glycoprotein H (Human herpes virus)
A
B
C
D
A: ROC curve B: Confusion matrix C: Ephrin type-A receptor 2(Human):envelope glycoprotein H(Human herpes virus) complex D: Open book conformation

## Slide 6
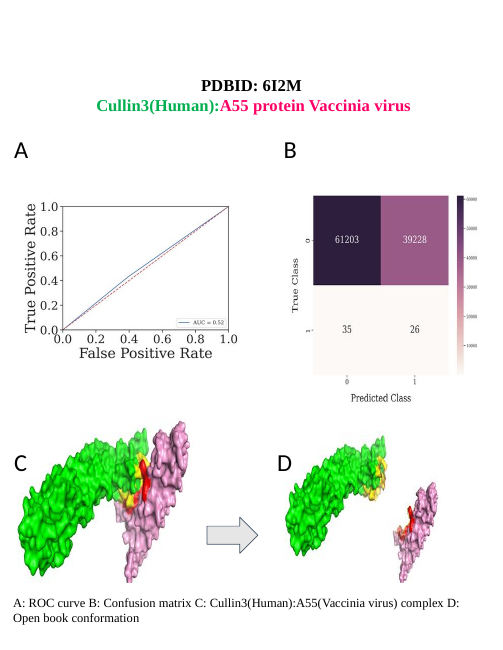

PDBID: 6I2M
Cullin3(Human):A55 protein Vaccinia virus
A
B
C
D
A: ROC curve B: Confusion matrix C: Cullin3(Human):A55(Vaccinia virus) complex D: Open book conformation

## Slide 7
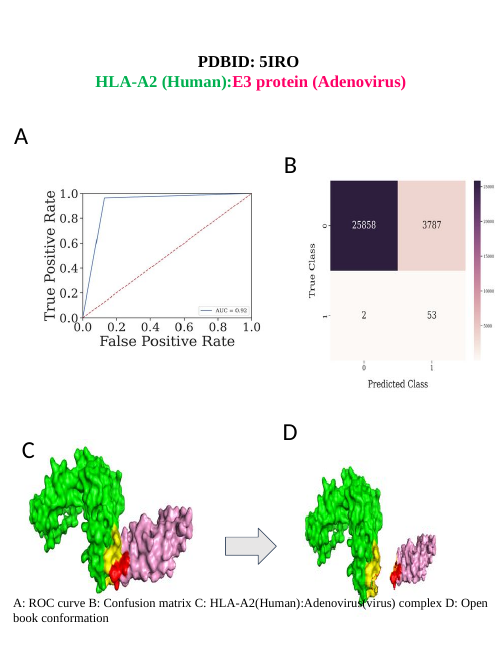

PDBID: 5IRO
HLA-A2 (Human):E3 protein (Adenovirus)
A
B
D
C
A: ROC curve B: Confusion matrix C: HLA-A2(Human):Adenovirus(virus) complex D: Open book conformation

## Slide 8
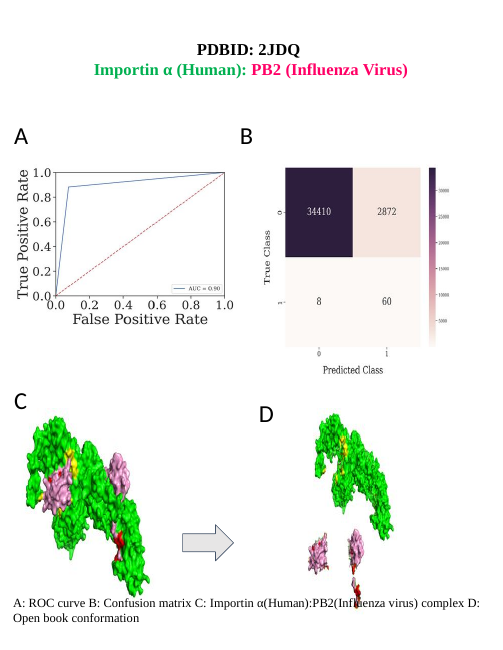

PDBID: 2JDQ
Importin α (Human): PB2 (Influenza Virus)
A
B
C
D
A: ROC curve B: Confusion matrix C: Importin α(Human):PB2(Influenza virus) complex D: Open book conformation

## Slide 9
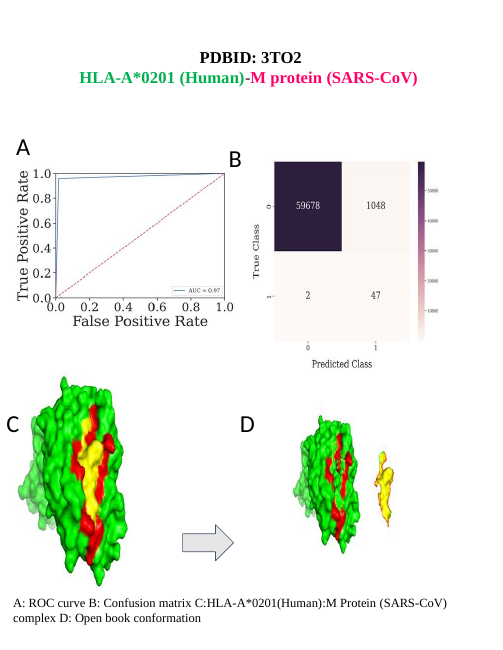

PDBID: 3TO2
HLA-A*0201 (Human)-M protein (SARS-CoV)
A
B
C
D
A: ROC curve B: Confusion matrix C:HLA-A*0201(Human):M Protein (SARS-CoV) complex D: Open book conformation

## Slide 10
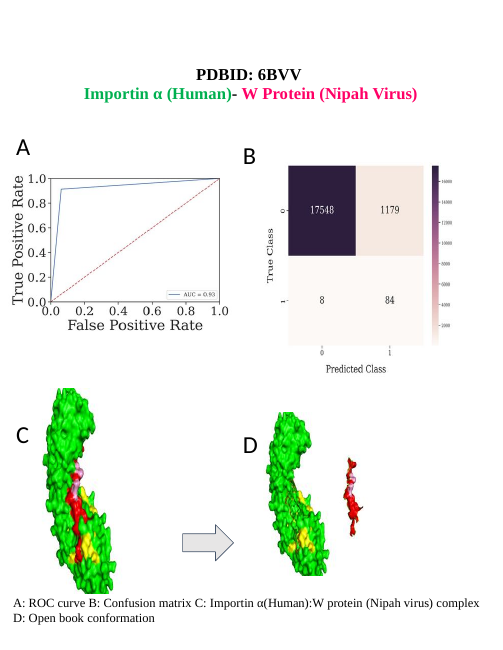

PDBID: 6BVV
Importin α (Human)- W Protein (Nipah Virus)
A
B
C
D
A: ROC curve B: Confusion matrix C: Importin α(Human):W protein (Nipah virus) complex D: Open book conformation

## Slide 11
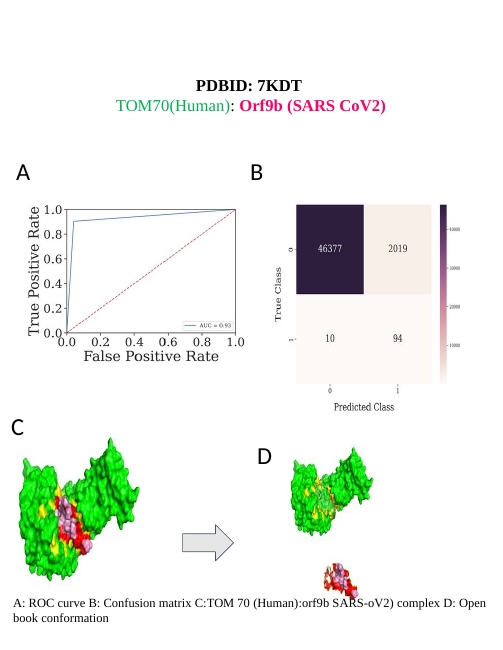

PDBID: 7KDT
TOM70(Human): Orf9b (SARS CoV2)
A
B
C
D
A: ROC curve B: Confusion matrix C:TOM 70 (Human):orf9b SARS-oV2) complex D: Open book conformation

## Slide 12
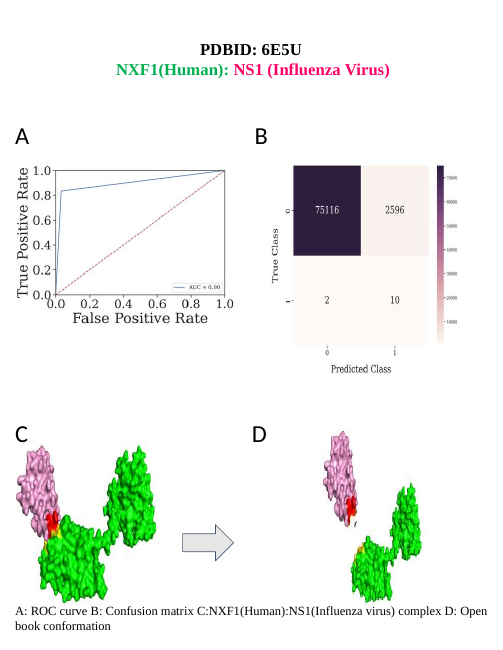

PDBID: 6E5U
NXF1(Human): NS1 (Influenza Virus)
A
B
C
D
A: ROC curve B: Confusion matrix C:NXF1(Human):NS1(Influenza virus) complex D: Open book conformation

## Slide 13
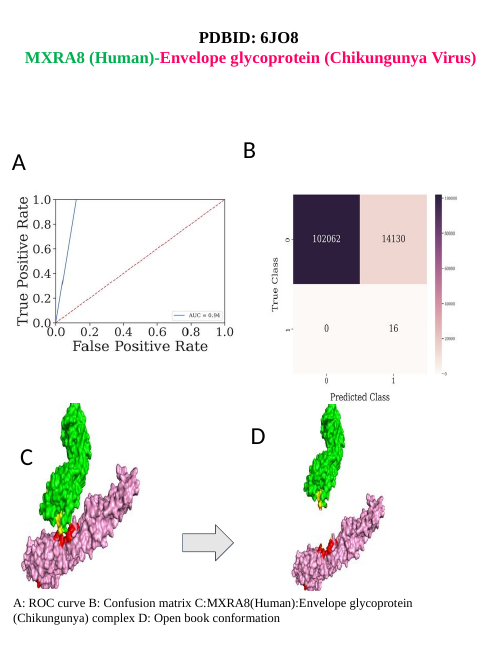

PDBID: 6JO8
MXRA8 (Human)-Envelope glycoprotein (Chikungunya Virus)
B
A
D
C
A: ROC curve B: Confusion matrix C:MXRA8(Human):Envelope glycoprotein (Chikungunya) complex D: Open book conformation

## Slide 14
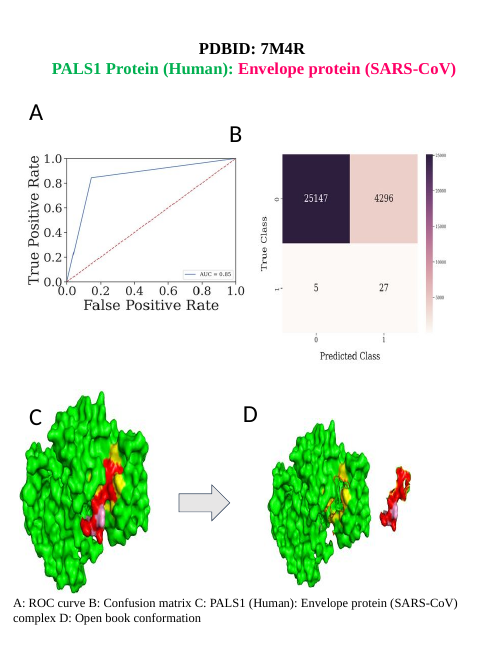

PDBID: 7M4R
PALS1 Protein (Human): Envelope protein (SARS-CoV)
A
B
D
C
A: ROC curve B: Confusion matrix C: PALS1 (Human): Envelope protein (SARS-CoV) complex D: Open book conformation

## Slide 15
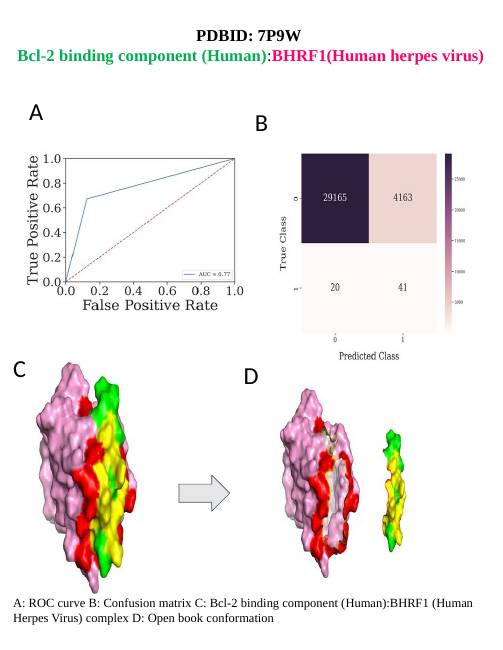

PDBID: 7P9W
Bcl-2 binding component (Human):BHRF1(Human herpes virus)
A
B
C
D
A: ROC curve B: Confusion matrix C: Bcl-2 binding component (Human):BHRF1 (Human Herpes Virus) complex D: Open book conformation

## Slide 16
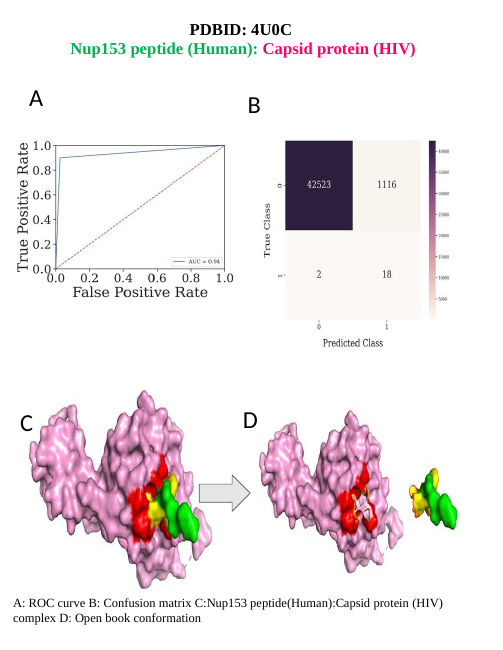

PDBID: 4U0C
Nup153 peptide (Human): Capsid protein (HIV)
A
B
D
C
A: ROC curve B: Confusion matrix C:Nup153 peptide(Human):Capsid protein (HIV) complex D: Open book conformation

## Slide 17
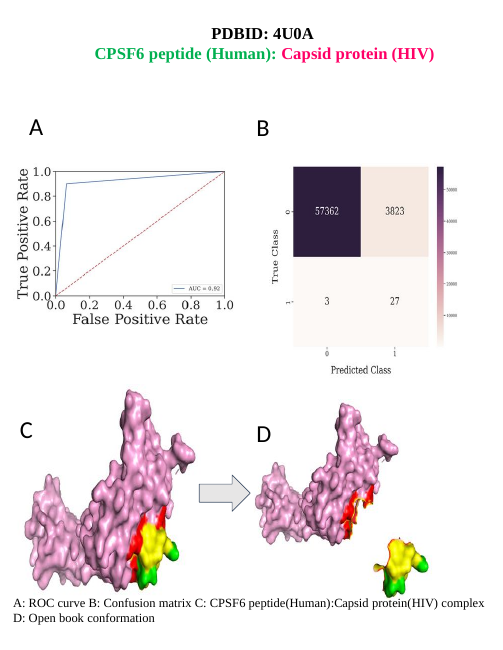

PDBID: 4U0A
CPSF6 peptide (Human): Capsid protein (HIV)
A
B
C
D
A: ROC curve B: Confusion matrix C: CPSF6 peptide(Human):Capsid protein(HIV) complex D: Open book conformation

## Slide 18
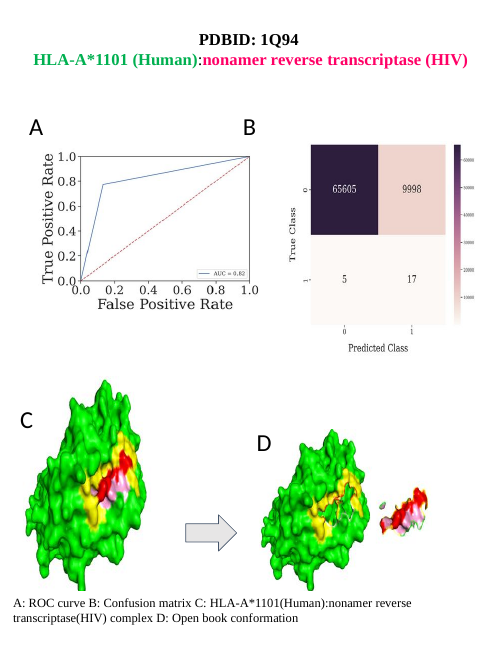

PDBID: 1Q94
HLA-A*1101 (Human):nonamer reverse transcriptase (HIV)
A
B
C
D
A: ROC curve B: Confusion matrix C: HLA-A*1101(Human):nonamer reverse transcriptase(HIV) complex D: Open book conformation
